# Engineering Dynamic Electrolyte Microenvironments via Double-Shell Hosts for Practical Lithium–Sulfur Batteries

**DOI:** 10.1007/s40820-026-02260-2

**Published:** 2026-06-22

**Authors:** Ziqing Yao, Yulu Zou, Shuqi Zhang, Wei Xie, Yujie Li, Shuangke Liu, Xingyu Chen, Kun Zhang, Chunman Zheng, Weiwei Sun

**Affiliations:** 1 College of Aerospace Science and Engineering, National University of Defense Technology, Changsha, 410073 People’s Republic of China; 2 School of Materials Science and Engineering, Changsha University of Science and Technology, Changsha, 410114 People’s Republic of China; 3 Department of Chemistry, National University of Singapore, Singapore, 117543 Singapore

**Keywords:** Lithium–sulfur batteries, Prussian blue analogues derivatives, Double-shell structure, Dynamic electrolyte microenvironments

## Abstract

The practical deployment of lithium–sulfur (Li–S) batteries is hampered by the shuttle effect of lithium polysulfides (LiPSs) and sluggish kinetics of sulfur redox reaction. While prevailing research focuses on maximizing the intrinsic activity of catalysts, the critical role of the host architecture in modulating the local reaction microenvironment remains overlooked. Herein, we report a facile ion-exchange strategy to synthesize double-shell hollow Prussian blue analogue derivative (Co_2.5_Fe/NC) as advanced sulfur hosts by precisely controlling the Co/Fe molar ratio at 2.5. Finite element simulations and in situ diagnostics reveal that the double-shell structure orchestrates a self-propelled electrolyte flow within the nanoreactor during operation. This dynamic flow effectively mitigates the spatial concentration heterogeneity of LiPSs, especially near the catalysts, thereby preventing active material passivation and ensuring sustained high catalytic efficiency. Consequently, even with a catalyst of moderate intrinsic activity, the Co_2.5_Fe/NC cathode achieves exceptional stability under lean electrolyte conditions (0.016% decay per cycle over 1000 cycles at 2 C). More importantly, an Ah-level pouch cell delivers a high energy density of 454.7 Wh kg⁻^1^. This work shifts the paradigm from solely pursuing catalytic activity to designing host structures that intelligently manage the electrochemical microenvironment, offering a new avenue toward practical Li–S batteries.

**Supplementary Information:**

The online version contains supplementary material available at 10.1007/s40820-026-02260-2.

## Highlights

- The efficient preparation of sulfur hosts derived from Prussian blue analogues with double-shell hollow structure was achieved through ion exchange with precisely controlled elemental ratios.
- The double-shell structure alters the microenvironment of sulfur redox reaction through self-driven dynamic migration of electrolyte, preventing the local enrichment of lithium polysulfides around catalysts.
- The double-shell sulfur cathode enhances the cycling stability and practical application of lithium–sulfur batteries, enabling an Ah-level pouch cell with an energy density of 454.7 Wh kg^–1^.

**Supplementary Information:**

The online version contains supplementary material available at 10.1007/s40820-026-02260-2.

## Graphical abstract

**Figure:**
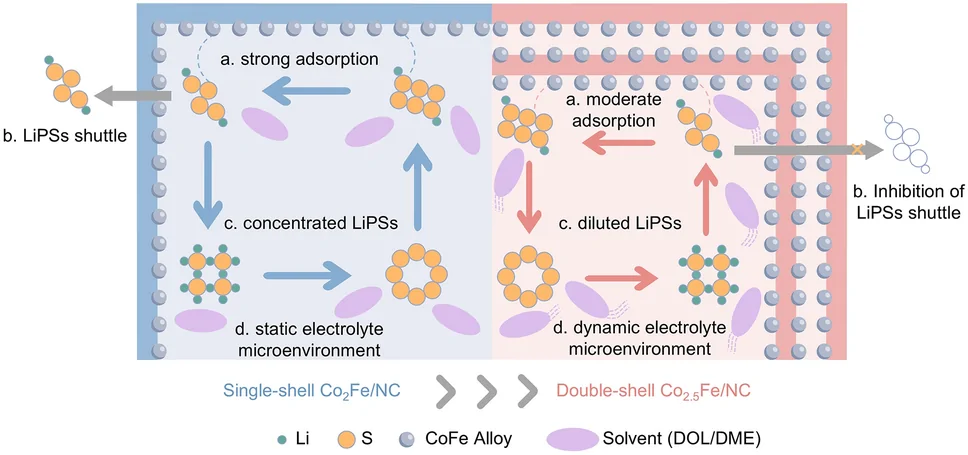

## Introduction

The relentless pursuit of higher energy density storage systems has positioned lithium–sulfur (Li–S) batteries at the forefront of next-generation battery technologies, owing to their exceptional theoretical specific energy (2600 Wh kg^⁻−1^) and the natural abundance of sulfur [1–4]. However, the process of transitioning from laboratory experimentation to commercial application is notoriously hindered by the inherent insulating properties of sulfur and lithium sulfide (Li_2_S), the fatal shuttling of soluble lithium polysulfides (LiPSs), and sluggish kinetics of the sulfur reduction reaction (SRR) [5–8]. To tackle these intertwined challenges, the research paradigm has overwhelmingly concentrated on engineering highly active electrocatalysts. A vast of catalytic materials, ranging from metallic compounds [9–11] and complex heterojunctions [12–14] to single-atom sites [15–17], etc., have been explored for the purpose of chemically adsorbing LiPSs and reducing the energy barriers for their conversion [18–20]. This catalyst-centric approach, while demonstrably effective, operates on the implicit assumption that the conductive host matrix plays a secondary role, confined solely to providing electron pathways and physical confinement [21–23].

**Fig. 1:**
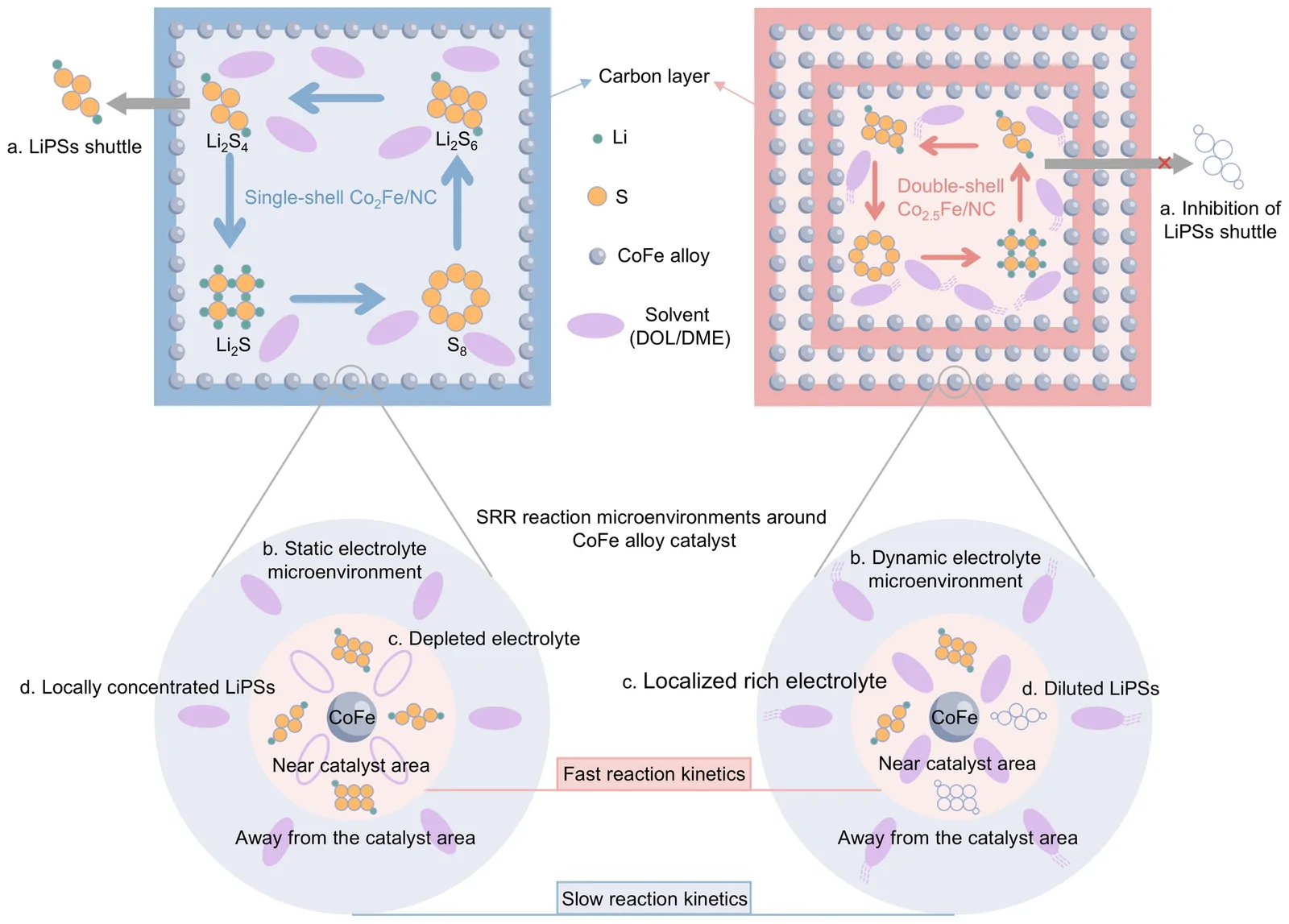
**a, b** Schematic illustration of the SRR reaction mechanism within a single-shell PBA derivative nanocube (**a**), with a static reaction microenvironment in the neighborhood of the catalyst (**b**). **c, d** Schematic illustration of the SRR reaction mechanism within a double-shell PBA derivative nanocube (**c**), with a dynamic reaction microenvironment in the neighborhood of the catalyst (**d**)

However, this prevailing focus neglects a fundamental electrochemical reality that emerges during battery operation. Within a confined nanoreactor, the accelerated reaction kinetics near a highly active catalyst, inevitably leads to rapid local consumption of reactants and the generation of high-concentration LiPSs [24, 25]. This process gives rise to severe spatial heterogeneity in concentration and electrolyte potential, which is analogous to the phenomenon of “local concentration polarization”. Under practical lean electrolyte conditions, such heterogeneity is exacerbated, leading to electrolyte depletion, LiPSs accumulation, and ultimately, the passivation of catalytic sites and formation of inactive “dead sulfur” [26]. Consequently, the full potential of an intrinsically superior catalyst may never be realized, as its local microenvironment becomes progressively poisoned. It can thus be concluded that the macroscopic architecture of the sulfur host is not merely a passive scaffold but rather a critical designer of the dynamic reaction microenvironment, a factor whose importance may rival, or even surpass, that of intrinsic catalytic activity in determining long-term, practical performance [27–29]. Reconceptualizing the host from a static framework to an active microenvironment regulator represents a pivotal yet underexplored frontier in Li–S battery design (Fig. 1).

Prussian blue analogues (PBAs), with their highly tunable composition and inherent hollow structures, emerge as an ideal model system to probe this structure–function relationship [30–33]. Their derivatives can be readily transformed into nitrogen-doped carbon matrices embedded with alloy nanoparticles, serving as integrated catalytic hosts [34–36]. Particularly, double-shell hollow structures offer superior specific surface area and more intricate internal space compared to their single-shell counterparts, hinting at enhanced capabilities for managing charge transport [37–39]. Yet, the precise mechanism by which such a multi-shell architecture modulates the internal electrochemical milieu during the dynamic SRR process remains elusive. Furthermore, a simplified, scalable synthetic route for such complex structures is desperately needed to facilitate fundamental study and practical application, moving beyond tedious and corrosive template-etching methods.

**Fig. 2:**
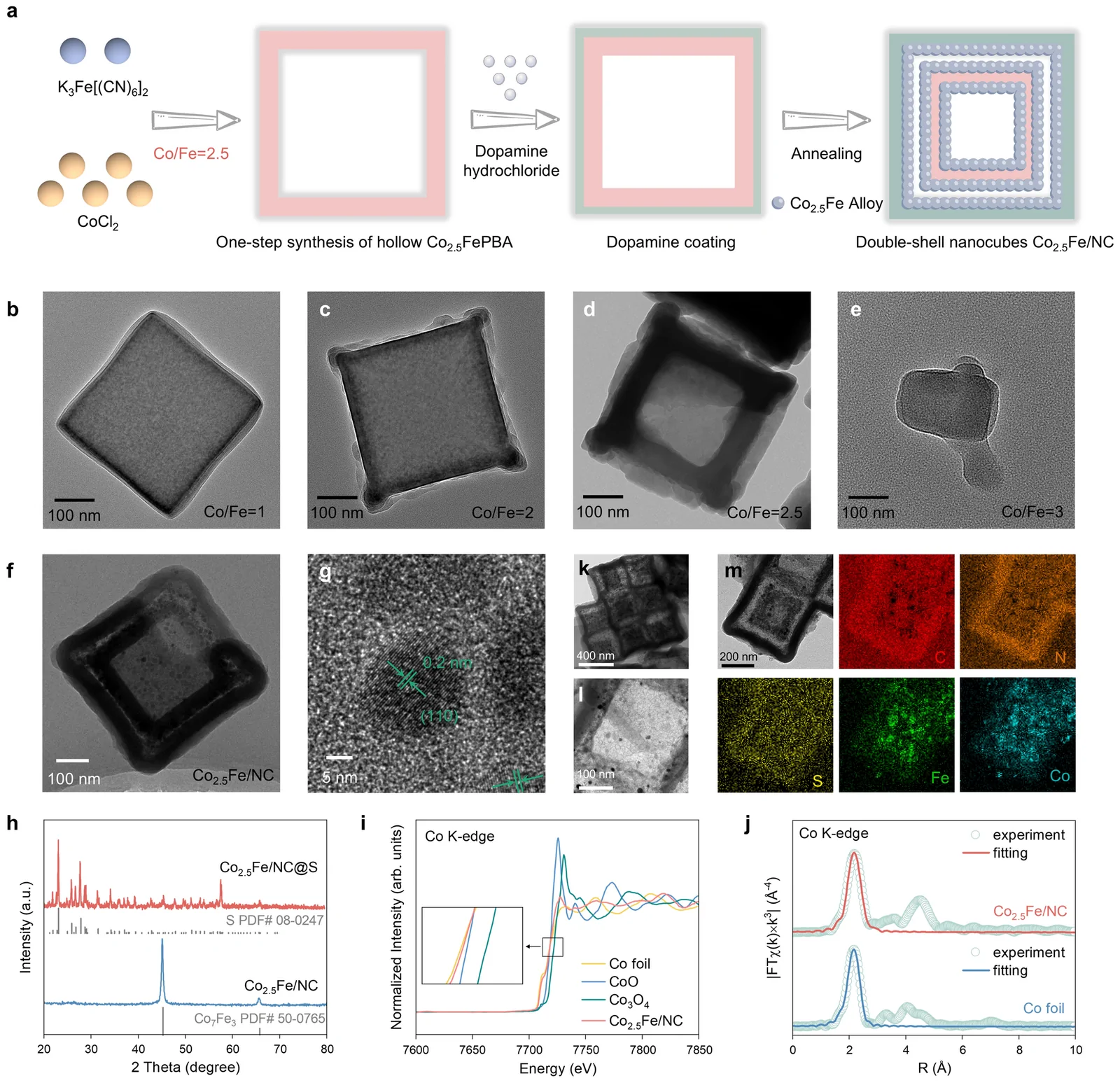
Controlled and simplified synthesis of double-shelled PBA derivatives and characterization of the morphology and structure. **a** Schematic diagram of the synthesis route for double-shell PBA derivatives loaded with Co_2.5_Fe alloy. **b–e** TEM morphology of CoFePBA nanocubes at different Co/Fe ratios (Co/Fe = 1, 2, 2.5, 3). **f** TEM image of Co_2.5_Fe/NC loaded with CoFe alloy. **g** High-resolution TEM image of Co_2.5_Fe alloy. **h** XRD patterns of Co_2.5_Fe/NC cathodes before and after sulfur fusion. **i** Co K-edge XANES of Co_2.5_Fe/NC. **j** Fourier transform magnitude of Co K-edge EXAFS spectrum of Co_2.5_Fe/NC and Co foil. **k, l** TEM images of the Co_2.5_Fe/NC@S cathodes after sulfur fusion. **m** Elemental EDS mapping of Co_2.5_Fe/NC@S

Herein, we demonstrate that the rational design of host architecture can intelligently engineer a self-improving reaction microenvironment, effectively decoupling long-term cycling stability from the sole pursuit of peak catalytic activity. We develop a facile, one-step ion-exchange strategy, strictly controlling the molar ratio of cobalt to iron salts at 2.5 for synthesizing double-shell hollow PBA derivatives (Co_2.5_Fe/NC), thereby avoiding complex etching processes. Through a combination of finite element simulations and in situ electrochemical diagnostics, we unveil that the double-shell structure uniquely induces a convective electrolyte flow within its cavity during operation. This dynamic microenvironment management actively mitigates local LiPSs concentration polarization around the catalysts, ensuring sustained reaction homogeneity and preventing active site passivation, as illustrated in Fig. 2. As a result, a cathode with a moderately active Co_2.5_Fe alloy catalyst dramatically outperforms one with a more active single-shell counterpart (Co_2_Fe/NC), achieving exceptional cycling stability (84.4% capacity retention after 1000 cycles at 2 C under lean electrolyte conditions) and a high energy density of 454.7 Wh kg^−1^ in an Ah-level pouch cell. This work shifts the design paradigm from a narrow focus on catalyst optimization to a holistic principle of “host-structure-induced microenvironment regulation,” providing a novel and essential perspective for developing practical high energy density Li–S batteries.

## Experimental Section

### Materials Preparation

Cobalt chloride hexahydrate (CoCl_2_·6H_2_O), Manganese(II) chloride tetrahydrate (MnCl_2_·4H_2_O), Nickel chloride hexahydrate (NiCl_2_·6H_2_O), ferric potassium cyanide (K_3_[Fe(CN)_6_]), sodium citrate dihydrate (HOC(COONa)(CH_2_COONa)_2_·2H_2_O), dopamine hydrochloride, tris(hydroxymethyl)aminomethane and sublimated sulfur were supplied by Aladdin company. Reagents are used directly without further purification.

*Synthesis of CoFePBA nanocubes:* Firstly, 1 mmol of K_3_[Fe(CN)_6_] was dissolved in 30 mL of deionized water to form Solution A. Separately, 1 mmol of CoCl_2_·6H_2_O and 2 mmol of sodium citrate dihydrate were dissolved in 30 mL of deionized water to form Solution B. The molar quantity of CoCl_2_·6H_2_O was modified to 2, 2.5, and 3 mmol in order to prepare samples with different cobalt-iron ratios. Solution A was slowly added to solution B and stirred vigorously on a magnetic stirrer for 5 min and then left to stand for 24h. The supernatant was poured off and the precipitate was washed three times with deionized water and ethanol and dried in an oven at 60 °C to obtain CoFePBA. MnFePBA and NiFePBA were prepared by replacing the CoCl_2_·6H_2_O mentioned above with MnCl_2_·4H_2_O and NiCl_2_·6H_2_O, respectively; the remaining steps were the same.

*Synthesis of CoFe/NC hollow nanocubes:* A buffer solution was prepared by dissolving 160 mg of tris(hydroxymethyl)aminomethane in 80 mL of deionized water, whereas 200 mg of preformed CoFePBA powder and 100 mg of dopamine hydrochloride were added to the buffer solution and centrifuged after 12 h of vigorous stirring and washed three times. The precipitate obtained was baked in a vacuum oven at 60 °C for 4 h to obtain dopamine coated CoFePBA powder. The powder was placed in a tube furnace and held under argon atmosphere at 500 °C for 2 h with a temperature increase rate of 2 °C min^−1^ to obtain the product as CoFe/NC.

*Synthesis of CoFe/NC@S:* Sulfur composite cathode was prepared by melt diffusion method. Specifically, CoFe/NC powder and sublimated sulfur were mixed thoroughly in a mass ratio of 1:5 and then placed in a tube furnace and heated in a N_2_ atmosphere at 155 °C for 12 h at a temperature increase rate of 2 °C min^−1^.

### Materials Characterization

The morphologies of the samples were characterized with a FEI Tecnai 12 BioTwin Transmission Electron Microscope (TEM) operated at 120 kV and a SU8000 Scanning Electron Microscope (SEM) at accelerating voltages from 3 keV down to 200 eV equipped with an Oxford Instruments Ultim Max detector for energy-dispersive spectroscopy (EDS). The crystal structures of the samples were characterized via ex situ XRD with Cu Kα radiation (λ = 1.5405 Å) by measuring the diffraction angle range of 10°-80°. Surface area data were collected on a Quadrasorb SI-MP gas sorption analyzer using ultrapure N_2_ (99.999%) and a liquid N_2_ bath. Brunauer–Emmett–Teller (BET) surface areas were determined by linear least squares regression analysis using the linearized form of BET equation. TGA data were obtained with a thermogravimetric analyzer (TGA600) to determine the mass content of each component in the composites. X-ray absorption fine structures (XAFS) of the Co K-edge and Fe K-edge were detected at the beamline BL14W1 in Shanghai Synchrotron Radiation Facility (SSRF) in the transmission mode. Energy calibration was performed by simultaneously measuring the spectra of a reference metal foil. The raw data were processed and analyzed using the Athena software package. Data process and analysis were carried out using the Demeter program pack with Athena.

### In Situ Raman Spectroscopic Measurements

Raman spectra were collected with Renishaw Raman microscopy. For in situ Raman spectroscopy, a custom cell mold with a quartz window in the negative case was used. CoFe/NC@S, Super P and aqueous acrylonitrile copolymer binder (commercial LA133 binder, Guangdong Canrd New Energy Technology Co., Ltd) were mixed well in a mass ratio of 8:1:1 and coated on Al meshs and dried under vacuum at 60 °C as the cathodes. A piece of lithium metal with a diameter of 16 mm and a thickness of 400 μm (99%, Guangdong Canrd New Energy Technology Co., Ltd) was used as the anode. A hole with a diameter of 0.4 mm was punched in the lithium foil to allow the laser to shine on the polypropylene separator (Celgard 2400). The thickness of separator was 25 μm, and the porosity was 41%. The electrolyte was 1 M lithium bis(trifluoromethanesulfonate)imide (LiTFSI) in DOL/DME (1:1 volume ratio), with 1 wt% LiNO_3_. The cells were assembled in an Ar-filled glove box (H_2_O < 0.01 ppm, O_2_ < 0.01 ppm). The cells were discharged at a current rate of 0.2 C from 1.7 to 2.8 V and tested at 25 °C. A low-magnitude × 50 objective was used, and Raman signals were recorded simultaneously with a 532 nm laser on the separator during the discharge process.

### Electrodes Fabrication and Cells Assembly

Sulfur composite cathodes were prepared by mixing CoFe/NC@S, SuperP and LA133 binder in the ratio of 80:12:8 by weight and an appropriate amount of deionized water, milled well to obtain a slurry, coated on a charcoal-coated Al foil collector, and then baked for 12 h at 60 °C in a vacuum oven. A 400 μm lithium foil was used as the anode and PP Celgard membrane as the separator was assembled with sulfur cathode as well as shrapnel and spacers to form a standard CR2025 coin cell in an Ar-filled glove box (H_2_O < 0.01 ppm, O_2_ < 0.01 ppm). The separator has a thickness of 25 microns, a diameter of 19 mm, and a porosity and average pore size of 55% and 64 nm, respectively. The electrolyte was 1 M LiTFSI in DOL/DME (1:1 volume ratio), with 1 wt% LiNO_3_. Li||CoFe/NC@S pouch cells were assembled by stacking a sulfur cathode and a 100 μm lithium foil. An Al tab is pressure-welded to the sulfur cathode, while a Ni tab is clamped to the lithium foil. The pouch cells were encapsulated using an Al-plastic film. Pouch cells are evacuated and sealed prior to liquid injection, and then electrolyte is injected through a syringe in a glove box and sealed a second time.

### Electrochemical Measurements

The charge/discharge test was conducted on a Neware battery testing system (CT-4008) in a specific potential range from 1.7 to 2.8 V. Ex situ/in situ EIS were collected with a perturbation of 5 mV in the frequency range from 1 MHz to 0.1 Hz on an electrochemical workstation (Zennium XC, Zahner). CV tests at different sweep rates were performed on an electrochemical workstation (Autolab PGSTAT-302N, Metrohm). The galvanostatic intermittent titration technique (GITT) test is conducted by charging/discharging at a current of 0.1 C for 5 min, followed by a 30 min relaxation period. The Li_2_S nucleation test was performed by discharging the cell at a constant current of 0.112 mA to 2.06 V and holding it at a constant potential of 2.05 V until the test current was less than 10^–5^ A. The desired Li_2_S_8_ was prepared by adding Li_2_S and S to DOL/DME (1:1 volume ratio) solvent in a molar ratio of 1:7 and stirring for 24 h at 60 °C to prepare Li_2_S_8_ solution (0.2 M). The battery was assembled in the gloved box, in which lithium foil worked as counter electrode, CoFe/NC as working electrode and 20 μL prepared Li_2_S_8_ solution was dropped onto CoFe/NC. Additionally, add 20 μL conventional electrolyte to the lithium anode.

### Computational Methods

The first-principles calculations were conducted by using Vienna Ab-initio Simulation Package (VASP) software with density functional theory (DFT) and projector augmented-wave plane-wave (PAW) pseudopotential method [40, 41]. The generalized gradient approximation (GGA) with the Perdew–Burke–Ernzerhof (PBE) function was applied to describe the electron exchange–correlation functions [42, 43]. The four crystal surface models (Co_1_Fe, Co_2_Fe, Co_2.5_Fe, Co_3_Fe) had a vacuum thickness of 15 Å in the z-direction to avoid interaction between the slabs. The kinetic energy cutoff of electron wave functions was used as 500 eV. The K-point meshes used for the first Brillouin zone integration were generated by Monkhorst–Pack scheme as 3 × 3 × 1. The energy band structure of each system was calculated by the closed K-point grid path of Γ-F-Q-Z-Γ. The DFT + U calculation method is adopted due to the strong correlation effect between the d electrons of metal atoms. A more accurate calculation is performed for the d orbitals of the transition metal atoms Co or Fe of the three structural models by setting a U value of 4 eV (Ueff = 4 eV). The energy convergence criterion for structural optimization of each model was set to 10^–4^ eV and the convergence criterion of atomic forces was set to 0.01 eV Å^−1^. To analyze the electronic structure, Crystal Occupation Hamilton Population (COHP) analysis was performed on LOBSTER software [44–46]. The adsorption energy (E_ads_) was used to measure the strength of the interaction between the surface of catalyst and sulfur species, and is calculated using the following formula:

1 $$ E_{{{\mathrm{ads}}}} = \, E_{{\text{(substrate and LiPSs)}}} {-} \, E_{{\mathrm{(substrate)}}} {-} \, E_{{\mathrm{(LiPSs)}}} $$

where E_(substrate and LiPSs)_ is the total energy of the adsorption configuration between the catalyst surface and LiPSs; E_(substrate)_ is the energy of the isolated catalyst surface; E_(LiPSs)_ is the energy of the isolated LiPSs. Reaction energy (ΔE) and reaction barrier energy (E_a_) of each step can be calculated according to the following formula:

2 $$ \Delta E \, = \, E_{{{\mathrm{FS}}}} - \, E_{{{\mathrm{IS}}}} $$

3 $$ E_{{\mathrm{a}}} = \, E_{{{\mathrm{TS}}}} {-} \, E_{{{\mathrm{IS}}}} $$

where E_IS_, E_TS_ and E_FS_ are the total energy of initial state, transition state and final state, respectively.

### Finite Element Simulation Methods

The models were calculated using some modules of COMSOL 6.3. A 2D multi-physics model integrating concentration, electric, and flow fields was established to compare the distributions of flow velocity, electric potential, and current density in the internal cavities of double-shell and single-shell structures. The double-shell configuration was defined with an overall size of 400 nm, a wall thickness of 30 nm, and an inter-shell spacing of 15 nm, while the single-shell structure shared the same overall size of 400 nm and wall thickness of 30 nm. For simplification, pore structures in the shell walls were neglected, and both shells were treated as homogeneous materials in the 2D simulation. A 3D model of a double-shell cavity with a porous surface was developed to simulate the concentration distribution of lithium polysulfides, comparing the molar concentration inside double-shell and single-shell cavities. Based on TEM images and BET testing, the following model parameters were approximated: The double-shell structure (400 nm size, 30 nm wall thickness, 15 nm inter-shell spacing) featured an average pore size of 12 nm, whereas the single-shell structure (400 nm size, 30 nm wall thickness) had an average pore size of 21 nm. In contrast to the 2D approach, the 3D model explicitly incorporated the actual porous structure. To determine the distribution of electrolyte flow velocity, potential, and current density within the nanocube, the following physical equations were employed:

- Mass transport (convection–diffusion in fluids)
  4 $$ \partial \left( {\varepsilon C} \right)/\partial t \, + \nabla \cdot( - D_{eff} \nabla C + uC) \, = \, R $$
  where u is flow velocity, R is source/sink term (e.g., chemical reactions) (mol m^−3^ s^−1^), C is species concentration, ϵ is porosity and D is diffusion coefficient.
- Fluid dynamics
  first equation is mass conservation (continuity equation):
  5 $$ \nabla \cdot\left( {\rho u} \right) \, = \, 0 $$
  second equation is momentum conservation (Navier–Stokes equations):
  6 $$ \rho (\partial u/\partial t \, + \, (u\cdot\nabla )u \, = \, - \nabla p \, + \, \mu \nabla^{2} u $$
  where u is velocity vector, p is pressure, μ is dynamic viscosity and ρ is density.
- Charge Conservation equation:
  This equation describes the conversation of energy:
  7 $$ \nabla \cdot \mathop k\limits^{ \to } = \, 0 $$
  8 $$ \vec{k}\; = - \sigma \nabla \varphi $$
  where ∇ is the divergence operator, $\overrightarrow{\mathrm{k}}$ is the total density vector (A m^−2^), σ is the effective ionic conductivity of the electrolyte (S m^−1^) and ∇φ is the gradient of the electric potential.

The coupled solution of the above equations yields the distributions of the electrical field, fluid velocity, and concentration field [38].

Finite element simulation was employed to numerically simulate the diffusion of LiPSs starting from an initial concentration of 100 mol m^−3^. The diffusion process was governed by Fick's law:

9 $$ \partial c/\partial t \, + \nabla \cdot ( - D \cdot \nabla c) \, = \, R $$

where c is the concentration, D the diffusion coefficient, and R the reaction rate. Since no reactions occur apart from diffusion, R was set to 0. Simulations track the evolution of LiPSs concentrations in single-shell and double-shell structures on time scales [47].

## Results and Discussion

### Simplified Fabrication and Characterization of Double-Shelled Hollow PBA Derivatives

The schematic synthesis route for preparing double-shell hollow PBA derivatives as high-performance sulfur host materials via ion-exchange regulation of the cobalt-iron ratio is illustrated in Fig. 2a. Specifically, cobalt chloride (CoCl_2_) was selected as the cobalt salt, and potassium ferrate (K_3_Fe[(CN)_6_]) as the iron salt. When the molar ratio of Co to Fe is precisely adjusted to 2.5, the one-step synthesis of a hollow structure of bimetallic CoFePBA can be achieved. As demonstrated by the CoFePBA morphology in Scanning Electron Microscopy (SEM, Fig. S1) and Transmission Electron Microscopy (TEM, Fig. 2d, e), when the cobalt-iron ratio was varied within the range of 1–3, both Co/Fe = 1 and Co/Fe = 2 exhibit well-defined solid nanocubes. At a composition of Co/Fe = 2.5, the material assembles into a characteristic hollow structure, with cube dimensions consistent with the previous two cases. Increasing the cobalt salt content to Co/Fe = 3 resulted in structural collapse, with the cubic morphology no longer being observed. The positions of the peaks in the X-ray diffraction (XRD) patterns of CoFePBA materials at different cobalt-iron ratios correspond to those in the PDF cards of the standard materials (Fig. S2), thereby confirming that this series of PBA materials share the same crystal structure. Figure S3 provides a visualization of the elemental distribution of the one-step synthesized hollow PBA through energy-dispersive X-ray spectroscopy (EDS) mapping. The results obtained reveal a uniform distribution of Co, Fe, C, and N elements across the nanocubic framework, with consistent microstructural dimensions throughout the product, all measuring 400 nm. The formation of a one-step hollow structure has been shown to be beneficial to the material with Co/Fe = 2.5 in the Brunauer–Emmett–Teller (BET) test (Fig. S4), which exhibits significantly higher specific surface area and porosity than CoFePBA materials with other ratios. It is interesting to note that analysis of the SEM images in Fig. S5 indicates that substituting cobalt salts with Co(NO_3_)_2_, or replacing iron salts with manganese or nickel salts, does not facilitate a one-step synthesis of hollow PBAs.

Subsequently, the one-step synthesized hollow CoFePBA is coated with dopamine hydrochloride, followed by heat treatment to achieve alloying of cobalt and iron elements and the formation of a double-shell structure. Figures 2f and S6 show the morphology and elemental distribution of the double-shelled nanocubes loaded with CoFe alloys (Co_2.5_Fe/NC). Their dimensions are comparable to those of the PBA precursor. The carbon and nitrogen elements aggregate at the double-shelled framework, while the cobalt and iron elements coalesce to form isolated nanoparticles within the structure. The presence of similar isolated nanoparticles has been detected at other Co/Fe ratios. However, single-shell nanocubes have been formed at Co/Fe = 1 and 2, while the structure has collapsed at Co/Fe = 3, thus exhibiting significant differences from the double-shell structure that has been observed at Co/Fe = 2.5 (Fig. S7). To investigate the composition of the internal nanoparticles, lattice fringes were observed using high-resolution transmission electron microscopy (HRTEM), as shown in Figs. 2g and S8, and it was found that their interplanar spacings were similar to those of the (110) and (200) planes of the cobalt-iron alloy. As illustrated in Fig. 2h, the XRD pattern of CoFe/NC exhibits peak positions that are identical to those of the cobalt-iron alloy, along with a peak intensity ratio that is remarkably consistent. This provides direct evidence that validates the composition of the catalyst as a CoFe alloy. X-ray absorption spectroscopy (XAS) can be employed to characterize the local coordination environment and valence state of elements. A thorough analysis of the extended X-ray absorption fine structure (EXAFS) at the Co K-edge, as shown in Fig. 2i, suggests that the valence state of Co approaches the zero-valence state that has been observed in Co foil. Figures 2j and S9, which present the Fourier transform amplitude and wavelet transform results of the Co K-edge EXAFS spectrum in CoFe/NC, along with the synchrotron radiation measurements of Fe K-edge in Fig. S10, corroborate this perspective [48]. A statistical analysis of the particle sizes within the TEM field of view was conducted, which revealed a typical normal distribution, with an average particle size of 7.76 nm (Fig. S11). This dimension is significantly smaller than the nanoreactor dimensions, allowing the assumption to be made that each particle exhibits broadly equivalent catalytic activity. In a manner analogous to that observed in CoFe PBA materials exhibiting variable ratios, the BET results presented in Fig. S12 indicate that the CoFe/NC material demonstrates the greatest specific surface area and pore volume at a Co/Fe ratio of 2.5. This phenomenon can be attributed to the unique double-shell structure of the lens. This advantage provides additional catalyst attachment sites and enhances the sulfur-hosting capacity for sulfur fusion. In order to systematically evaluate the performance of CoFe/NC materials at varying ratios as sulfur hosts within the Li–S batteries, molten diffusion was employed to permeate elemental sulfur into different host matrices. Thermogravimetric analysis (TGA) revealed an effective sulfur loading of 82.54% within the final CoFe/NC@S cathode composite (Fig. S13). Concurrently, EDS elemental mapping verified the uniform distribution of sulfur within the cavity.

The one-step synthesis of hollow PBA was achieved by regulating the molar ratio of Co salt to Fe salt at 2.5. The creation of a double-shell structure loaded with alloy catalysts was achieved through a process involving dopamine hydrochloride coating and thermal treatment. In contrast, single-shell structures were obtained under different conditions. Utilizing a series of products with varying ratios of Co/Fe as sulfur hosts (Co_1_Fe/NC, Co_2_Fe/NC, Co_2.5_Fe/NC, and Co_3_Fe/NC), among which Co_2.5_Fe/NC exhibits a unique double-shell structure, we conducted further investigation into the differences in their electrochemical performance, as well as the mechanisms by which catalysts and morphological structures influence the cathodic reaction of sulfur.

### Electrochemical Performance of PBA Derivatives in Li–S Batteries

To evaluate the electrochemical performance of PBA derivatives, they were thoroughly fused with sulfur at a 1:5 mass ratio. The resulting mixture was then cast to form a cathode comprising CoFe/NC@S, conductive carbon (Super P), and a water-soluble polymer-lauryl acrylate (LA133) binder in a mass ratio of 80:12:8 (see details in the Experimental Section). The cathode was subsequently assembled with a lithium foil anode to form a coin cell, which was then subjected to electrochemical evaluation.

**Fig. 3:**
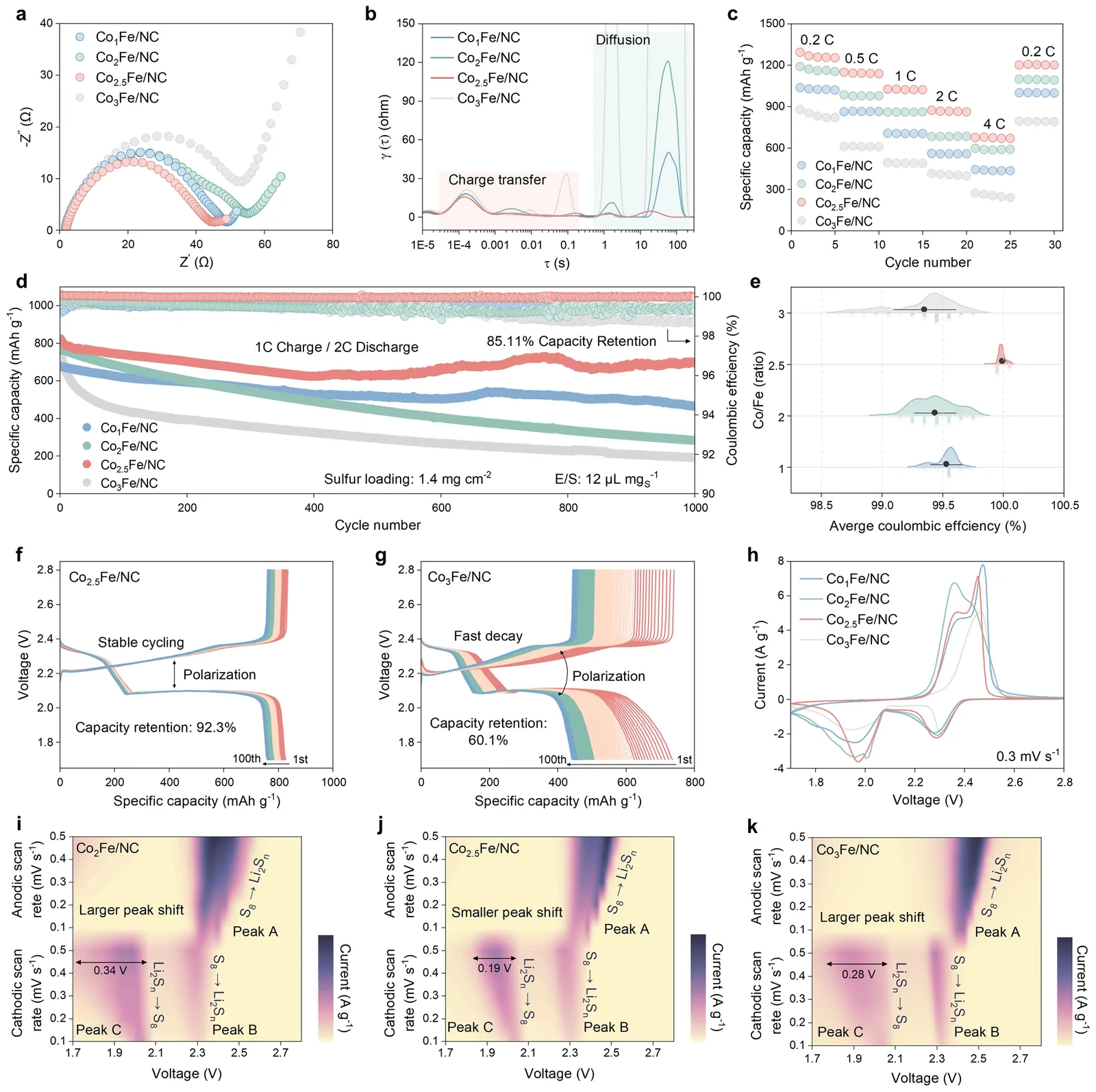
Electrochemical performance of CoFe/NC cathodes with different Co/Fe ratios. EIS spectra of **a** different CoFe/NC cathodes and **b** corresponding DRT analysis. **c** Rate performance of different batteries. **d** Long-term cycling performance of different batteries at 2 C current density under lean electrolyte conditions. **e** Violin plots of coulombic efficiency for different cathodes over long cycles. **f, g** Charge–discharge curves for the best-performing Co_2.5_Fe/NC cathode (f) and the worst-performing Co_3_Fe/NC cathode during the first 100 cycles. **h** CV curves of different batteries at a scan rate of 0.3 mV s^−1^. Contour plots of CV patterns for **i** Co_2_Fe/NC, **j** Co_2.5_Fe/NC, and **k** Co_3_Fe/NC with different scan rates

In Li–S batteries, the conductivity of the sulfur cathode is typically poor, which leads to significant polarization. The incorporation of carbon materials as a sulfur host has been demonstrated to enhance conductivity and reduce polarization. Electrochemical impedance spectroscopy (EIS) coupled with distribution of relaxation time (DRT) analysis is an effective method of evaluating battery conductivity [49, 50]. As demonstrated in Fig. 3a, b, Co_2.5_Fe/NC demonstrates the lowest charge transfer impedance and diffusion impedance, attributable to its double-shell carbon layer, which enhances both electron and lithium-ion transport capabilities. Unfortunately, Co_3_Fe/NC is susceptible to structural collapse-induced agglomeration, resulting in significantly increased charge transfer and diffusion impedances. This has the effect of impeding capacity delivery under conditions of high current densities.

As illustrated in Fig. 3c, the capacity discharge of the four cathodes is shown to range from 0.2 to 4 C (1 C = 1675 mA g^−1^). It is evident that the Co_2.5_Fe/NC cathode exhibits a high specific capacity of 1294.25 mAh g^−1^ at 0.2 C, indicating high sulfur utilization. Notably, even at a high current density of 4 C, the battery exhibits an exceptional reversible capacity of 676.42 mAh g^−1^, thereby demonstrating a substantially augmented rate performance in comparison to the other three cathodes. The relative rate performance of different cathodes is found to correlate with the differences in electron and ion transport capabilities, as revealed by impedance analysis.

Furthermore, the long cycling performance of the batteries at a high current of 2 C was tested, with the lean electrolyte condition maintained at an electrolyte/sulfur (E/S) ratio of approximately 12. As was the case with its performance at the same rate, Co_2.5_Fe/NC cathode exhibited the highest initial discharge capacity among the four cathodes, achieving a specific capacity of 823.42 mAh g^−1^. Following 1000 cycles, the Co_2.5_Fe/NC cathode demonstrated a capacity of 700.84 mAh g^−1^, exhibiting an impressive capacity retention rate of 84.4% and excellent cycling stability. As shown in Table S1, Co_2.5_Fe/NC exhibits a competitive advantage in terms of cycling stability and rate capability among the reported sulfur cathodes. However, the remaining three cathodes exhibited significant disparities in both cycling stability and capacity retention. It is noteworthy that the Co_3_Fe/NC cathode, despite attaining a comparatively elevated initial discharge specific capacity of 734.84 mAh g^−1^, underwent accelerated capacity degradation during the initial 200 cycles, ultimately retaining a mere 26.09% of its initial capacity. The capacity release of sulfur cathodes is typically governed by the activity of the catalyst, which in turn determines the efficiency with which sulfur is utilized. Nevertheless, the stability of the system during cycling is contingent on the interplay of numerous factors, including the morphology of the sulfur host. Coulombic efficiency is indicative of the reversibility of the charging and discharging processes. Figure 3e presents the violin plots of Coulombic efficiency for batteries assembled with four distinct cathodes over 1000 cycles. As anticipated, the Co_2.5_Fe/NC cathode exhibited an average coulombic efficiency of 99.95%, demonstrating stability throughout the cycling process. Conversely, the Co_3_Fe/NC cathode demonstrated high dispersion in coulombic efficiency, with an average value of 99.35%. High Coulombic efficiency is indicative of superior reversibility in electrochemical reactions, which in turn implies a reduced occurrence of undesirable phenomena such as LiPSs shuttling and consequent loss of active sulfur. The charge–discharge curves for the initial 100 cycles of the two cathodes that exhibited the highest and lowest performance levels have been selected for further analysis, as illustrated in Fig. 3f, g. In the context of Co_2.5_Fe/NC cathodes, the discharge curves observed across varying cycle numbers manifest distinct dual discharge plateaus. The initial plateau, occurring at approximately 2.38 V, accounts for around one-quarter of the total discharge capacity, while the subsequent plateau, occurring at around 2.12 V, accounts for three-quarters of the total discharge capacity. For Co_3_Fe/NC cathodes, an increase in the number of cycles results in a gradual blurring of the dual-platform structure of the discharge curve, accompanied by a rapid decline in specific capacity. This phenomenon indicates a substantial loss of active material. Furthermore, the degree of polarization in Co_3_Fe/NC cathodes increases substantially, whereas the polarization in Co_2.5_Fe/NC cathodes remains negligible with cycling.

Cyclic voltammetry (CV) was next conducted to investigate the sulfur reduction kinetics at the electrode, employing scan rates ranging from 0.1 to 0.5 mV S^−1^ within the potential window of 1.7 to 2.8 V. As illustrated in Fig. 3h, the CV curves of various cathodes at a constant scan rate exhibit discernible variations in the positions of the oxidation peaks. The peak voltage of Co_2_Fe is lower than that of others, indicating a smaller overpotential and higher catalytic activity. Line graphs at all scanning rates are displayed in Fig. S14. In order to facilitate a more intuitive comparison of the differences between the various cathodes, Figs. 3i-k and S15 illustrate contour plots in CV mode. The anodic peak (Peak A), which is observed at approximately 2.32 V during anodic scanning, is attributed to the transformation of Li_2_S to LiPSs and S_8_. Conversely, the two cathodic peaks, which are observed at ~ 2.35 V (Peak B) and ~ 2.09 V (Peak C) during cathodic scanning, correspond, respectively, to the reduction of S_8_ to LiPSs and LiPSs to Li_2_S. In terms of peak width analysis, it was found that the peaks A, B, and C of the Co_2.5_Fe/NC cathode exhibited the smallest peak shift with varying scan rates, particularly Peak C, which showed a shift of merely 0.19 V. Their significantly narrower width compared to the other three cathodes indicates superior reversibility, consistent with the coulombic efficiency results. On the other hand, analysis of the peak current magnitude provides insights into the electrode diffusion process. The classical Randles–Sevcik equation is applied to describe lithium-ion diffusion:

10 $$ I_{p} = \, \left( {2.69 \times 10^{5} } \right)n^{1.5} SD_{Li + }^{0.5} C_{Li + } v^{0.5} $$

In this equation, I_p_ denotes the peak current, n represents the charge transfer number, S is the geometric area of the active electrode, D_Li+_ is the lithium-ion diffusion coefficient, C_Li+_ is the lithium-ion concentration within the cathode, and v is the potential scanning rate [51, 52]. The slope of the curve (I_p_/v^0.5^) indicates the lithium-ion diffusion rate. The gradient of the curve presented in Fig. S16 is indicative of the lithium-ion diffusion rate, given the constant values of n, S and C. It is evident that the Co_3_Fe/NC cathode demonstrates the worst performance, attributable to its weaker catalytic properties and collapsed carbon framework structure. The Co_2_Fe/NC and Co_2.5_Fe/NC cathodes demonstrate comparable excellent performance, though whether this stems from superior catalytic properties or dynamic reaction optimization conferred by the double-shell structure warrants further investigation. The Co_2.5_Fe/NC cathode demonstrates a remarkably high slope, indicating fast SRR reaction kinetics. However, whether this advantage stems from the intrinsic enhanced activity of the alloy catalyst or unique optimization provided by the double-shell structure requires further investigation.

### DFT Calculation of Alloy Catalyst Activity and Simulation of Carbon Framework Functionality

**Fig. 4:**
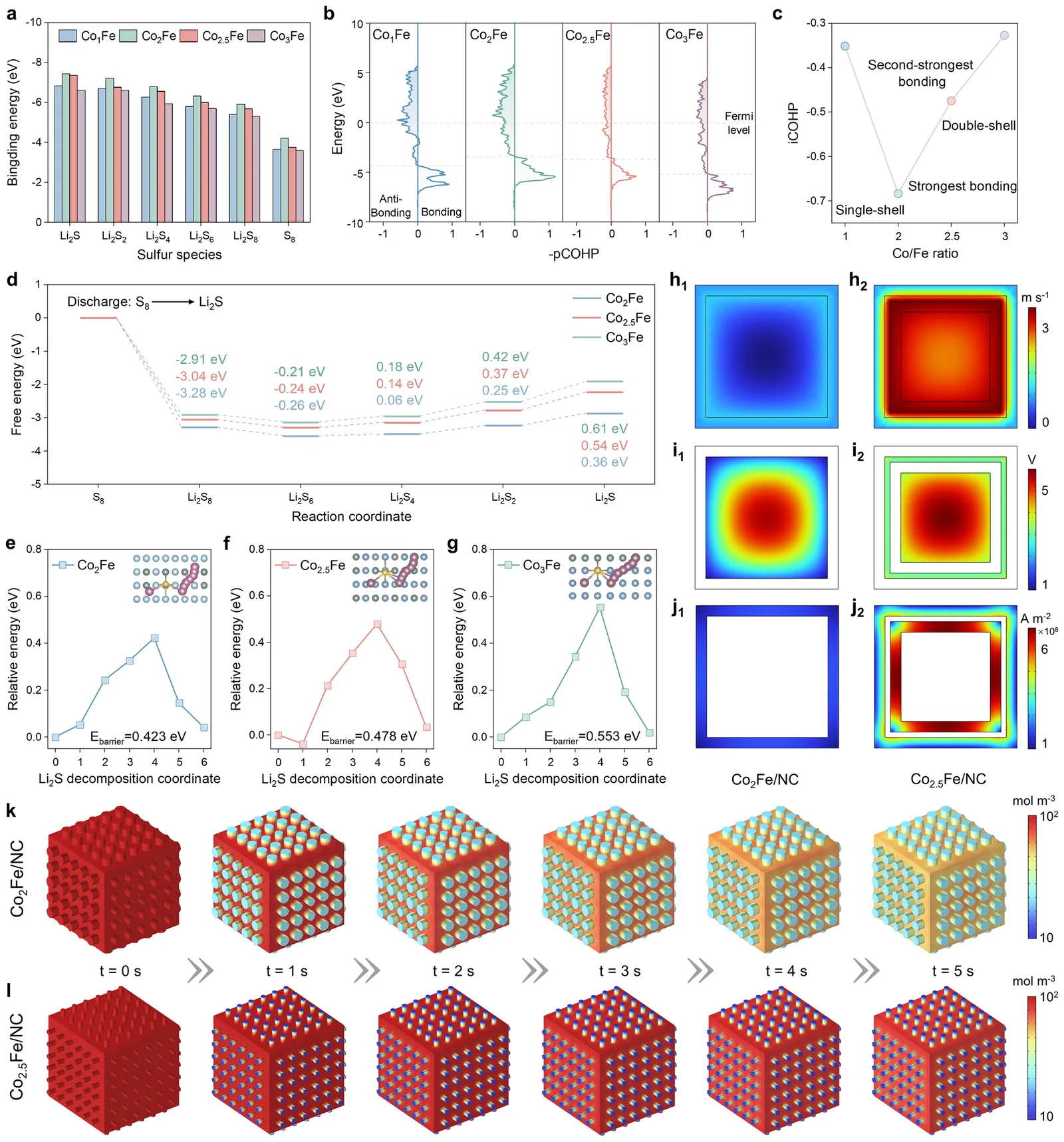
DFT calculation of catalytic activity for CoFe alloys at varying Co/Fe ratios and finite element simulation of the mechanism governing single/double-shell structures. **a** Binding energies of S_8_/Li_2_S_n_ species on different cathodes. **b** COHPs and **c** iCOHPs of Li_2_S_4_ adsorbed on the different CoFe alloys. **d** Gibbs free energy on various catalysts.—Decomposition energy barriers of Li_2_S on **e** Co_2_Fe, **f** Co_2.5_Fe and **g** Co_3_Fe. **h** Analysis of electrolyte flow rates inside and on the surface of Co_2_Fe/NC (h_1_) and Co_2.5_Fe/NC (h_2_) during the reaction. **i** Surface electrolyte potential analysis of Co_2_Fe/NC (i_1_) and Co_2.5_Fe/NC (i_2_) in electrochemical reactions. **j** Analysis of electrolyte current density on the surface of Co_2_Fe/NC (j_1_) and Co_2.5_Fe/NC (j_2_) in electrochemical reactions. **k, l** Three-dimensional finite element simulations of Li_2_S_4_ diffusion in single-shell Co_2_Fe/NC cathode and double-shell Co_2.5_Fe/NC cathode (initial concentration is 100 mol m^−3^ at t = 0 s)

The comprehensive electrochemical performance of composite sulfur cathodes is attributable to the synergistic interaction between the catalyst, which modulates the LiPSs conversion kinetics, and the carbon materials. In order to investigate the activity of alloy catalysts with varying cobalt-iron ratios, a series of proportional models were constructed and subjected to DFT calculations. The catalyst provides active sites for the conversion of sulfur species through chemical adsorption; its strong adsorption capacity captures LiPSs, thereby preventing the shuttle effect. As demonstrated in Fig. S17, a clear distinction in adsorption energies is evident across the three discrete adsorption sites on the cobalt-iron alloy catalyst. The findings suggest that sulfur species exhibit a greater propensity to bind to the CoFe dual site, thereby demonstrating the superiority of this alloy catalyst. Furthermore, the adsorption energy for LiPSs is observed to vary in accordance with the varying proportions of CoFe elements within the four cathodes, as illustrated in Fig. 4a. Figure S18 presents the schematic diagrams illustrating the binding of Li_2_S_4_ to the catalyst. A thorough analysis of the binding energy indicates that the Co_2_Fe catalyst exhibits the strongest binding energy for a series of sulfur species, signifying the most potent adsorption capacity for LiPSs. This finding is inconsistent with the optimal electrochemical performance demonstrated by the Co_2.5_Fe/NC cathode.

The predicted and integrated crystal orbital Hamiltonian population (pCOHP and iCOHP of bonds formed between catalysts and intermediates provides an intuitive reflection of bond strength. Li_2_S_4_, a pivotal reaction intermediate, demonstrates elevated solubility, which is the principal factor contributing to the shuttle effect [53]. Moreover, the high energy barrier for the liquid–solid conversion reaction from Li_2_S_4_ to Li_2_S is the primary factor contributing to the sluggish SRR kinetics. Consequently, Li_2_S_4_ is typically selected as a representative LiPS species for investigation. From Co_2_Fe to Co_3_Fe, the antibonding states of the bond between catalyst and Li_2_S_4_ shift further downwards below the Fermi level and become filled, indicating a weakened bond. The same applies to Co_1_Fe (Fig. 4b). A similar conclusion can be drawn by comparing the iCOHP values: the closer the iCOHP approaches zero, the weaker the bond becomes, potentially nearing fracture, as shown in Fig. 4c.

The Sabatier principle emphasizes that the binding strength between the catalyst and reactants must be moderate to achieve optimal catalytic activity. This suggests that contrary to popular belief, binding energy is not necessarily better the higher it is. Instead, there exists a threshold beyond which catalytic activity actually diminishes, thereby giving rise to the well-known “volcano” relationship. However, the question of whether the Co_2_Fe catalyst with the highest binding energy has reached this threshold remains a subject of debate. To this end, Gibbs free energy calculations were performed for the transformation of S_8_ to Li_2_S during the discharge process as illustrated in Fig. 4d. Given the observation that the binding energies of Co_1_Fe and Co_3_Fe catalysts exhibit analogous behavior, this study proceeded to conduct further theoretical calculations using the Co_3_Fe/NC cathode undergoing structural transformation as a representative example. The Gibbs free energy change has been shown to provide an intuitive reflection of the catalyst's efficacy in enhancing the kinetics of the SRR reaction. The results demonstrate a close alignment with the pattern exhibited by the binding energy in Fig. 4a, with Co_2_Fe once again demonstrating the most favorable performance, Co_3_Fe the least favorable, and Co_2.5_Fe exhibiting an intermediate performance. Moreover, as presented in Fig. 4d, the decomposition energy barrier of Li_2_S during charging also align with this correspondence, with the performance ranking from best to worst being Co_2_Fe, Co_2.5_Fe, and Co_3_Fe, respectively.

The trend in SRR kinetic regulation by alloy catalysts with varying elemental ratios has been shown to correlate with the binding energy of catalysts and intermediates. This implies that the Co₂Fe catalyst, possessing the highest binding energy, does not reach the threshold of the Sabatier principle. Consequently, the Co_2_Fe alloy exhibits superior adsorption capacity, thereby suppressing the severe shuttle effect of LiPSs. Simultaneously, it demonstrates optimal regulatory capability over SRR kinetics. The Co_2_Fe/NC cathode is hypothesized to demonstrate optimal electrochemical performance, encompassing rate capability, capacity delivery, and cycling stability, due to these two significant advantages. However, the reality is that the Co_2_Fe/NC cathode exhibits significantly superior electrochemical performance compared to the Co_2_Fe/NC cathode, particularly in terms of capacity retention under long-cycle conditions.

In comparison to Co_2_Fe/NC and Co_2.5_Fe/NC cathodes, significant disparities emerge in their morphological structures, beyond the variation in catalyst: The composition of Co_2_Fe/NC has been shown to be single-shelled nanocubes, while Co_2.5_Fe has been found to feature a double-shelled structure. This structural disparity in nanoreactors may provide a rationale for the discrepancy between the activity of catalyst and the electrochemical performance of these cathodes within Li–S batteries.

To investigate the question of whether single-shell and double-shell structures influence the electrochemical reactions at sulfur cathodes and to elucidate the underlying mechanisms, finite element simulation techniques were employed. The modeling was conducted on the basis of material characterization, specifically the physical properties of size and pore diameter, as illustrated in Fig. S19. A detailed simulation was conducted utilizing the COMSOL software to explore the flow of electrolyte within and on the surface of nanoreactors for both Co_2_Fe/NC and Co_2.5_Fe/NC cathodes, incorporating varying numbers of shell layers (see details in Supporting Information). The finite element simulations unravel a fundamental distinction: the double-shell architecture establishes a convective electrolyte flow (unit: m s^−1^) within the cavity, whereas electrolyte remains largely stagnant in the single-shell counterpart (Fig. 4h_1_, h_2_). This induced convection actively transports polysulfides away from the catalyst surface, alleviating localized concentration buildup and ensuring a more uniform reaction field. Furthermore, the electrolyte potential (unit: V) governing the spatial distribution of nanocubes during electrochemical reactions was analyzed and simulated (Fig. 4i_1_, i_2_), and the current density (unit: A m^−2^) on the carbon framework loaded with catalyst within nanoreactors featuring two distinct shell structures was evaluated (Fig. 4j_1_, j_2_). A series of results indicate that the double-shell hollow structure exhibits higher overall reaction potential and current density, with particularly elevated reactivity at the edges and apexes, thereby accelerating reaction kinetics. It can be deduced that this is attributable to the dynamic electrolyte within the double-shell structure, which entrains the high-concentration LiPSs generated near the catalyst as it flows through the reactor. As a result of this process, spatial inhomogeneity in reactivity within the reactor is mitigated, albeit to a certain extent.

Moreover, the three-dimensional simulation results reveal that the concentration of LiPSs at the pore ends of the double-shell structure is significantly lower as the reaction progresses over time, indicating its effective capability to restrict the escape of soluble LiPSs from the interior of the nanoreactor and thereby prevent the shuttle effect. The suppression of the shuttle effect is partly attributable to the chemical adsorption of LiPSs by Co_2.5_Fe/NC. More significantly, the double-shell framework facilitates internal electrolyte flow, enabling uniform distribution of LiPSs. This prevents localized overconcentration that would otherwise trigger concentration diffusion toward the exterior of the nanocubes. The double-shell structure of nanoreactor has been shown to induce dynamic migration of electrolyte within the structure, thereby compensating for the inherent activity limitations of the Co_2.5_Fe/NC cathode catalyst. This results in superior overall SRR reaction kinetics while firmly confining LiPSs within the double-shell framework, effectively suppressing the shuttle effect. Consequently, Hence, the discrepancy between the optimal electrochemical performance of the Co_2.5_Fe/NC cathode and its relatively poor catalytic activity is reasonably explained.

### In Situ Diagnostics and Practical Pouch Cell Validation

**Fig. 5:**
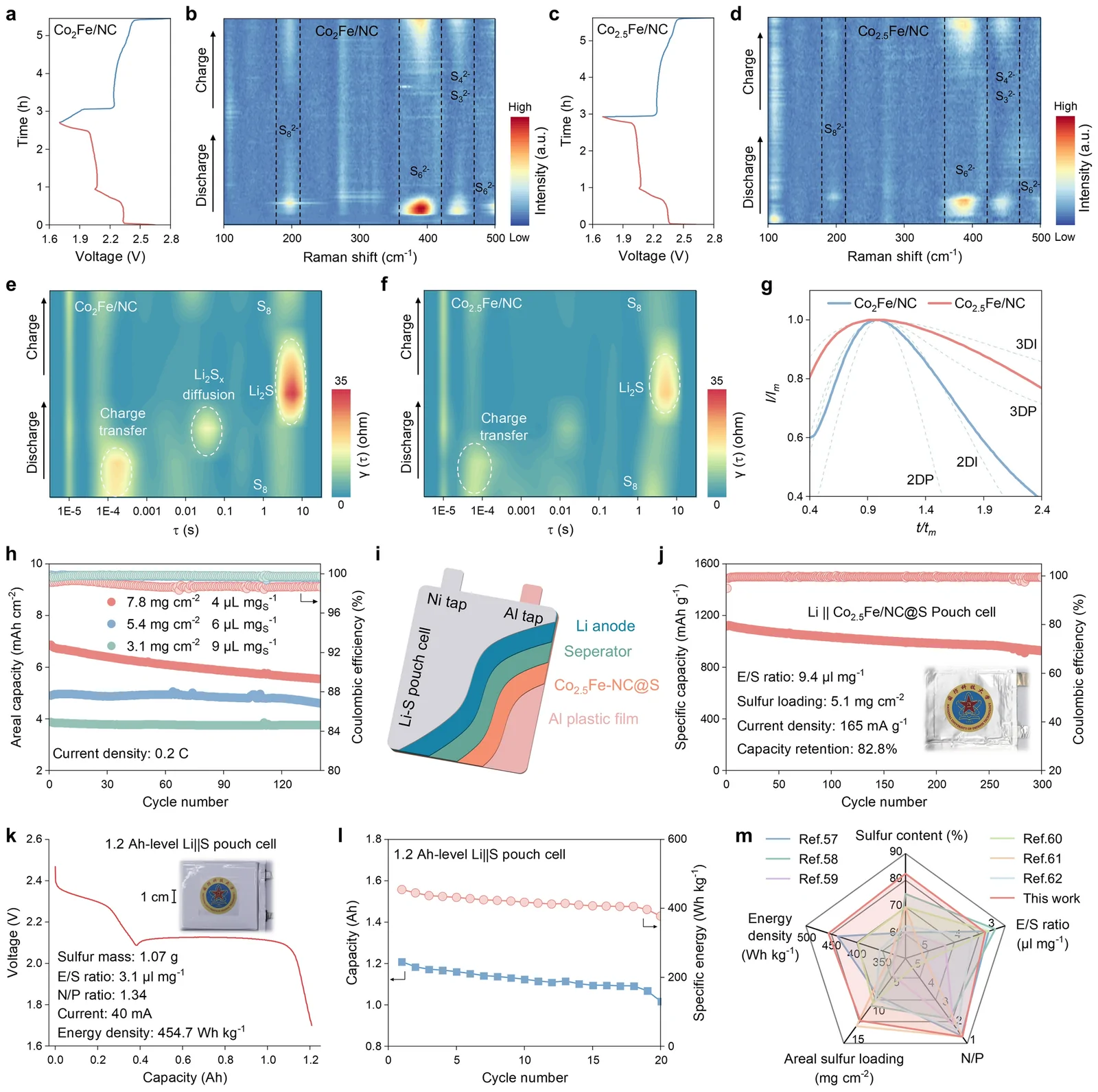
In situ diagnostics and performance verification of practical Li–S batteries. **a** Charge and discharge curves and **b** corresponding in situ Raman contour plot for Co_2_Fe/NC cathode. **c** Charge and discharge curves and **d** corresponding in situ Raman contour plot for Co_2.5_Fe/NC cathode. Contour plots of DRT curves from in situ EIS tests of **e** Co_2_Fe/NC and **f** Co_2.5_Fe/NC. **g** Dimensionless current–time transients of different cathodes to perform peak fitting according to theoretical 2D and 3D models. **h** Cycling performance of Co_2.5_Fe/NC cathode under high sulfur loading and lean electrolyte conditions. **i** Schematic diagram of the structure of a stacked-type Li–S pouch cell. **j** Cycling performance and photographs of the small pouch cell. **k** Photograph and discharge profile of an Ah-level pouch cell, which delivers a high density of 454.7 Wh kg^−1^. **l** Cycling performance of the Ah-level pouch cell. **m** Radar plot for performance comparison between this work and previously reported Li–S pouch cells

The analysis employs DFT calculations and finite element simulations to elucidate the intrinsic mechanism whereby the double-shell structure of the Co_2.5_Fe/NC cathode compensates for its inherent weakness in catalytic activity by inducing electrolyte flow within the nanoreactor. In order to provide further experimental evidence for the substantial advantages of the Co_2.5_Fe/NC cathode's double-shell structure, in situ Raman spectra of both cathodes were examined during charge–discharge cycles to analyze their impact on the LiPSs shuttle effect (Figs. 5a–d and S20). When analyzing the diffusion of LiPSs to the lithium anode side, only a small amount of signal from S_6_^2−^species (~ 400 cm^−1^) was detected during discharge and charging with the Co_2.5_Fe/NC cathode, demonstrating a combined improvement of the shuttle effect (Fig. 5d). On the contrary, stronger signals of S_6_^2−^ and attributed to S_4_^2−^ + S_3_^2−^ (~ 450 cm^−1^) were detected at the Co_2_Fe/NC cathode with a single-shell layer, implying the presence of severe LiPSs, causing the loss of active sulfur and bringing about capacity degradation (Fig. 5b).

Within the single-shell structure of Co_2_Fe/NC cathodes, the pronounced spatial inhomogeneity facilitates the formation of locally concentrated LiPSs environments near the catalyst. Conversely, in Co_2.5_Fe/NC cathodes, the flowing electrolyte rapidly dilutes the LiPSs generated around the catalyst. In situ EIS provides detailed insights into this process, while the corresponding DRT analysis captures and decouples this dynamic and complex process. As demonstrated in the Nyquist plot of in situ EIS (Fig. S21), Co_2.5_Fe/NC exhibits significantly lower impedance compared to Co_2_Fe/NC and greater stability during charge–discharge process. The DRT contour maps offer a more detailed comparison of the two cathodes (Fig. 5e, f). It should be noted that the comparatively elevated diffusion impedance observed at the beginning and termination of both charging and discharging is attributable to the inadequate ionic conductivity of Li_2_S and S_8_. However, the presence of a double-shell carbon layer in Co_2.5_Fe/NC has been shown to mitigate this intrinsic impedance. Crucially, high charge transfer impedance (τ ≈ 8 μs) and LiPSs diffusion impedance (τ ≈ 0.07 μs) were detected in Co_2_Fe/NC, indicating the formation of enriched LiPSs in the electrolyte, which in turn impedes further ion and electron transport [54]. When electron transport is restricted, subsequent slow kinetics arise due to the inhibition of further conversion reactions of sulfur species. In contrast, the Co_2.5_Fe/NC cathode exhibits not only significantly weaker charge transfer resistance but also virtually no discernible LiPSs diffusion impedance. This demonstrates that the double-shell structure enhances spatial homogeneity within the nanoreactor via mobile electrolyte, preventing localized high concentrations of LiPSs and mitigating electrochemical and concentration polarization during the reaction process. The results of the galvanostatic intermittent titration technique (GITT) for the two cathodes in Fig. S22 also confirm the faster ion transport rate and lower internal resistance in Co_2.5_Fe/NC.

Owing to its superior structural design, the Co_2.5_Fe/NC cathode exhibits a higher Li_2_S nucleation efficiency in the discharge product nucleation curve shown in Fig. S23, indicating a greater discharge capacity. To decipher the distinct growth behaviors of Li_2_S, a dimensionless diagnostic analysis of the current–time curves for Li_2_S nucleation was performed using the Scharifker–Hills model (Fig. 5g). Here, 3DI and 3DP denote instantaneous (I) and progressive (P) nucleation of three-dimensional (3D) hemispherical nuclei governed by ion diffusion rates, while 2DI and 2DP represent two-dimensional (2D) growth mechanisms controlled by lattice insertion (see detail in Supporting Information). The nucleation mechanism of Li_2_S evolves from a 2DI to a 3D growth pattern intermediate between 3 and 3DP, as the cathode evolves from a single-shell to a double-shell configuration. This indicates that the double-shell structure promotes instantaneous nucleation and forms uniform, dense Li_2_S deposits [55, 56].

The superior double-shell structure of the Co_2.5_Fe/NC cathode was leveraged in order to further evaluate its electrochemical properties under conditions of high areal loading sulfur cathodes and depleted electrolyte. Ah-level pouch cells were assembled in order to validate its applicability under operational conditions. As illustrated in Fig. 5h, the areal capacity performance of the Co_2.5_Fe/NC cathode at a current density of 0.2 C is demonstrated, with sulfur loadings of 3.1, 5.4, and 7.8 mg cm^−2^, and E/S ratios of 9, 6 and 4 μL mg^−1^, respectively. When the sulfur areal loading reaches 3.1 mg cm^−2^, the areal capacity of battery has been shown to exceed that of conventional intercalation cathodes. As the sulfur load of 3.1 and 5.4 mg cm^−2^, there was no statistically significant change in the areal capacity after 140 cycles. Notably, even under conditions of elevated sulfur load (7.8 mg cm^−2^), the material exhibited a notable areal capacity of 5.53 mAh cm^−2^ after cycling.

The achievement of pouch cells with energy densities in excess of 400 Wh kg^−1^ is contingent on the presence of high sulfur loading cathodes and low electrolyte usage. The pouch cell structure employing Co_2.5_Fe/NC cathode, separator and 50 μm lithium foil stacked sequentially is illustrated in Fig. 5i. The fabricated low-capacity pouch cell exhibited a specific capacity discharge of 1119.14 mAh g^−1^ at a current density of 0.1 C, demonstrating stable cycling performance over 300 cycles with a capacity decay of 0.057% per cycle (Fig. 5j). Further assembly of Ah-level Li–S batteries employing Co_2.5_Fe/NC cathodes, as illustrated in Fig. 5k, demonstrated a pouch cell with a total sulfur loading of 1.07 g (with 5 layers of cathode and 6 layers of anode) delivering over 1.2 Ah capacity. Utilizing a low E/S ratio (E/S = 3.1 μL mg^−1^) and negative / positive (N/P) ratio (N/P = 1.34), the battery achieved an exceptionally high energy density of 454.7 Wh kg^−1^ (without considering the weight of pouching case, tabs and labels). In consideration of the weight of all the weight of pouch cell, the calculated energy density of pouch cell is as high as 380.5 Wh kg^−1^ (details are presented in Table S2). This Ah-level pouch cell exhibited stable cycling for nearly 20 cycles at a current density of 40 mA g^−1^ under demanding conditions (Fig. 5l). Figure 5m shows that the double-shelled Co_2.5_Fe/NC, synthesized via a simplified method and employed as the sulfur host material, exhibits significantly superior performance parameters compared to previously reported Li–S batteries [57–62]. This validates the rationality of the synergistic enhancement of the SRR integrated reaction mechanism through the catalytic function of catalyst and the unique advantage of double-shell structure, namely inducing dynamic migration of electrolyte to prevent excessive local LiPSs concentration.

## Conclusions

In summary, we have demonstrated that the electrochemical microenvironment, governed by the host structure, is a decisive factor which is often overshadowed by the pursuit of catalytic activity in Li–S batteries. Through a combination of tailored synthesis, finite element simulation, and in situ electrochemical diagnostics, we elucidate that a double-shell hollow architecture can actively engineer a dynamic electrolyte flow to homogenize the concentration of LiPSs and electric potential field, mitigating localized catalyst poisoning and ensuring sustained reaction kinetics. As a direct result, the cathode with a moderately active catalyst but superior microenvironment management (Co_2.5_Fe/NC) exhibits significantly superior performance in comparison to its counterpart with higher intrinsic activity but a static single-shell structure. Our work underscores the paradigm of “host structure-induced microenvironment regulation” as a powerful design principle for next-generation sulfur hosts, paving the way for the development of Li–S batteries with high energy density and extended lifespan.

## Supplementary Information

Below is the link to the electronic supplementary material.

Supplementary file1 (DOCX 7737 KB)
