# Supplementary material for: Engineering Dynamic Electrolyte Microenvironments via Double-Shell Hosts for Practical Lithium–Sulfur Batteries
Source: Nanomicro Lett. 2026 Jun 22;18:406. doi: 10.1007/s40820-026-02260-2 (PMC13283951; doi:10.1007/s40820-026-02260-2)
Supplement: Supplementary file 1 — Supplementary file1 (DOCX 7737 KB) [file 40820_2026_2260_MOESM1_ESM.docx]

Supporting Information for

**Engineering Dynamic Electrolyte Microenvironments via Double-Shell Hosts for Practical Lithium-Sulfur Batteries**

Ziqing Yao^1#^, Yulu Zou^1#^, Shuqi Zhang^1^, Wei Xie^2^, Yujie Li^1^, Shuangke Liu^1^, Xingyu Chen^1^, Kun Zhang^3^, Chunman Zheng^1,^ *, and Weiwei Sun^1,^ *

^1^ College of Aerospace Science and Engineering, National University of Defense Technology, Changsha 410073, P. R. China

^2^ School of Materials Science and Engineering, Changsha University of Science and Technology, Changsha 410114, P. R. China

^3^ Department of Chemistry, National University of Singapore, Singapore 117543, Singapore

#Ziqing Yao and Yulu Zou contributed equally to this work.

*Corresponding authors. E-mail: zhengchunman@nudt.edu.cn (Chunman Zheng); wwsun@nudt.edu.cn (Weiwei Sun)

# S1 Experimental Section

# S1.1 Materials preparation

Cobalt chloride hexahydrate (CoCl_2_·6H_2_O), ferric potassium cyanide (K_3_[Fe(CN)_6_]), sodium citrate dihydrate (HOC(COONa)(CH_2_COONa)_2_·2H_2_O), dopamine hydrochloride, tris(hydroxymethyl)aminomethane and sublimated sulfur were supplied by Aladdin company. Reagents are used directly without further purification.

**Synthesis of CoFePBA nanocubes:** Firstly, 1 mmol of K_3_[Fe(CN)_6_] was dissolved in 30 ml of deionized water to form Solution A. Separately, 1 mmol of CoCl_2_·6H_2_O and 2 mmol of sodium citrate dihydrate were dissolved in 30 ml of deionized water to form Solution B. The molar quantity of CoCl_2_·6H_2_O was modified to 2 mmol, 2.5 mmol and 3 mmol in order to prepare samples with different cobalt-iron ratios. Solution A was slowly added to solution B and stirred vigorously on a magnetic stirrer for 5 minutes, and then left to stand for 24 hours. The supernatant was poured off and the precipitate was washed three times with deionized water and ethanol and dried in an oven at 60 ^o^C to obtain CoFePBA.

**Synthesis of CoFe/NC hollow nanocubes:** A buffer solution was prepared by dissolving 160 mg of tris(hydroxymethyl)aminomethane in 80 ml of deionized water, whereas 200 mg of preformed CoFePBA powder and 100 mg of dopamine hydrochloride were added to the buffer solution and centrifuged after 12 h of vigorous stirring and washed three times. The precipitate obtained was baked in a vacuum oven at 60 ^o^C for 4 hours to obtain dopamine coated CoFePBA powder. The powder was placed in a tube furnace and held under argon atmosphere at 500 ^o^C for 2 h with a temperature increase rate of 2 ^o^C min^-1^ to obtain the product as CoFe/NC.

**Synthesis of CoFe/NC@S:** Sulfur composite cathode was prepared by melt diffusion method. Specifically, CoFe/NC powder and sublimated sulfur were mixed thoroughly in a mass ratio of 1:5, and then placed in a tube furnace and heated in a N_2_ atmosphere at 155 ^o^C for 12 hours at a temperature increase rate of 2 ^o^C min^-1^.

# S1.2 Materials characterization

The morphologies of the samples were characterized with a FEI Tecnai 12 BioTwin Transmission Electron Microscope (TEM) operated at 120 kV and a SU8000 Scanning Electron Microscope (SEM) at accelerating voltages from 3 KeV down to 200 eV equipped with a Oxford Instruments Ultim Max detector for energy dispersive spectroscopy (EDS). The crystal structures of the samples were characterized via ex situ XRD with Cu Kα radiation (λ = 1.5405 Å) by measuring the diffraction angle range of 10-80^o^. Surface area data were collected on a Quadrasorb SI-MP gas sorption analyzer using ultrapure N_2_ (99.999%) and a liquid N_2_ bath. Brunauer-Emmett-Teller (BET) surface areas were determined by linear least squares regression analysis using the linearized form of BET equation. TGA data were obtained with a thermogravimetric analyzer (TGA600) to determine the mass content of each component in the composites. X-ray absorption fine structures (XAFS) of the Co K-edge and Fe K-edge were detected at the beamline BL14W1 in Shanghai Synchrotron Radiation Facility (SSRF) in the transmission mode. Energy calibration was performed by simultaneously measuring the spectra of a reference metal foil. The raw data were processed and analyzed using the Athena software package. Data process and analysis were carried out using the Demeter program pack with Athena.

# S1.3 In situ Raman spectroscopic measurements

Raman spectra were collected with Renishaw Raman microscopy. For in situ Raman spectroscopy, a custom cell mould with a quartz window in the negative case was used. CoFe/NC@S, Super P and aqueous acrylonitrile copolymer binder (commercial LA-133 binder, Guangdong Canrd New Energy Technology Co., Ltd) were mixed well in a mass ratio of 8:1:1 and coated on Al meshs and dried under vacuum at 60 ^o^C as the cathodes. A piece of lithium metal with a diameter of 16 mm and a thickness of 400 μm (99%, Guangdong Canrd New Energy Technology Co., Ltd) was used as the anode. A hole with a diameter of 0.4 mm was punched in the lithium foil to allow the laser to shine on the polypropylene separator (Celgard 2400). The thickness of separator was 25 μm, and the porosity was 41%. The electrolyte was 1 M lithium bis trifluoromethanesulfonate imide (LiTFSI) in DOL/DME (1:1 volume ratio), with 1 wt% LiNO_3_. The cells were assembled in an Ar-filled glove box (H_2_O < 0.01 ppm, O_2_ < 0.01 ppm). The cells were discharged at a current rate of 0.2 C from 1.7 V to 2.8 V and tested at 25 °C. A low-magnitude ×50 objective was used, and Raman signals were recorded simultaneously with a 532 nm laser on the separator during the discharge process.

# S1.4 Electrode fabrication and cells assembly

Sulfur composite cathodes were prepared by mixing CoFe/NC@S, SuperP and LA133 binder in the ratio of 80:12:8 by weight and an appropriate amount of deionized water, milled well to obtain a slurry, coated on a charcoal-coated Al foil collector, and then baked for 12 h at 60 ^o^C in a vacuum oven. A 400 μm lithium foil was used as the anode and PP Celgard membrane as the separator was assembled with sulfur cathode as well as shrapnel and spacers to form a standard CR2025 coin cell in an Ar-filled glove box (H_2_O < 0.01 ppm, O_2_ < 0.01 ppm). The separator has a thickness of 25 microns, a diameter of 19 mm, and a porosity and average pore size of 55% and 64 nm, respectively. The electrolyte was 1 M LiTFSI in DOL/DME (1:1 volume ratio), with 1 wt% LiNO_3_. Li||CoFe/NC@S pouch cells were assembled by stacking a sulfur cathode and a 100 μm lithium foil. An Al tab is pressure-welded to the sulfur cathode, while a Ni tab is clamped to the lithium foil. The pouch cells were encapsulated using an Al-plastic film. Pouch cells are evacuated and sealed prior to liquid injection, then electrolyte is injected through a syringe in a glove box and sealed a second time.

# S1.5 Electrochemical measurements

The charge/discharge test was conducted on a Neware battery testing system (CT-4008) in a specific potential range from 1.7 V to 2.8 V. Ex situ/in situ EIS were collected with a perturbation of 5 mV in the frequency range from 1 MHz to 0.1 Hz on an electrochemical workstation (Zennium XC, Zahner). CV tests at different sweep rates were performed on an electrochemical workstation (Autolab PGSTAT-302N, Metrohm). The galvanostatic intermittent titration technique (GITT) test is conducted by charging/discharging at a current of 0.1C for 5 minutes, followed by a 30 minutes relaxation period. The Li_2_S nucleation test was performed by discharging the cell at a constant current of 0.112 mA to 2.06 V and holding it at a constant potential of 2.05 V until the test current was less than 10^-5^A. The desired Li_2_S_8_ was prepared by adding Li_2_S and S to DOL/DME (1:1 volume ratio) solvent in a molar ratio of 1:7 and stirring for 24 h at 60 ^o^C to prepare Li_2_S_8_ solution (0.2 M). The battery was assembled in the gloved box, in which lithium foil worked as counter electrode, CoFe/NC as working electrode and 20 μL prepared Li_2_S_8_ solution was dropped onto CoFe/NC. Additionally, add 20 μL conventional electrolyte to the lithium anode.

# S1.6 Computational methods

The first-principles calculations were conducted by using Vienna Ab-initio Simulation Package (VASP) software with density functional theory (DFT) and projector augmented-wave plane-wave (PAW) pseudopotential method.[S1, S2] The generalized gradient approximation (GGA) with the Perdew-Burke-Ernzerhof (PBE) function was applied to describe the electron exchange-correlation functions.[S3, S4] The four crystal surface models (Co_1_Fe, Co_2_Fe, Co_2.5_Fe, Co_3_Fe) had a vacuum thickness of 15 Å in the z-direction to avoid interaction between the slabs. The kinetic energy cutoff of electron wave functions was used as 500 eV. The K-point meshes used for the first Brillouin zone integration were generated by Monkhorst-Pack scheme as 3 × 3 × 1. The energy band structure of each system was calculated by the closed K-point grid path of Γ-F-Q-Z-Γ. The DFT+U calculation method is adopted due to the strong correlation effect between the d electrons of metal atoms. A more accurate calculation is performed for the d orbitals of the transition metal atoms Co or Fe of the three structural models by setting a U value of 4 eV (Ueff=4 eV). The energy convergence criterion for structural optimization of each model was set to 10^-4^ eV and the convergence criterion of atomic forces was set to 0.01 eV Å^-1^. To analyze the electronic structure, Crystal Occupation Hamilton Population (COHP) analysis was performed on LOBSTER software.[S5–S7] The adsorption energy (E_ads_) was used to measure the strength of the interaction between the surface of catalyst and sulfur species, and is calculated using the following formula:

E_ads_ = E_(substrate and LiPSs)_ – E_(substrate)_ – E_(LiPSs)_

where E_(substrate and LiPSs)_ is the total energy of the adsorption configuration between the catalyst surface and LiPSs; E_(substrate)_ is the energy of the isolated catalyst surface; E_(LiPSs)_ is the energy of the isolated LiPSs. Reaction energy (ΔE) and reaction barrier energy (E_a_) of each step can be calculated according to the following formula:

ΔE = E_FS_ - E_IS_

E_a_ = E_TS_ – E_IS_

where E_IS_, E_TS_ and E_FS_ are the total energy of initial state, transition state and final state, respectively.

# S1.7 Finite element simulation methods

The models were calculated using some modules of COMSOL 6.3. A 2D multi-physics model integrating concentration, electric, and flow fields was established to compare the distributions of flow velocity, electric potential, and current density in the internal cavities of double-shell and single-shell structures. The double-shell configuration was defined with an overall size of 400 nm, a wall thickness of 30 nm, and an inter-shell spacing of 15 nm, while the single-shell structure shared the same overall size of 400 nm and wall thickness of 30 nm. For simplification, pore structures in the shell walls were neglected, and both shells were treated as homogeneous materials in the 2D simulation. A 3D model of a double-shell cavity with a porous surface was developed to simulate the concentration distribution of lithium polysulfides, comparing the molar concentration inside double-shell and single-shell cavities. The double-shell structure (400 nm size, 30 nm wall thickness, 15 nm inter-shell spacing) featured an average pore size of 12 nm, whereas the single-shell structure (400 nm size, 30 nm wall thickness) had an average pore size of 21l nm. In contrast to the 2D approach, the 3D model explicitly incorporated the actual porous structure. To determine the distribution of electrolyte flow velocity, potential, and current density within the nanocube, the following physical equations were employed:

a) mass transport (convection-diffusion in fluids)

∂(ϵC)/∂t + ∇·(-D_eff_∇C+uC)=R

where u is flow velocity, R is source/sink term (e.g., chemical reactions) [mol/(m^3^ s)], C is species concentration, ϵ is porosity and D is diffusion coefficient.

b) fluid dynamics

first equation is mass conservation (continuity equation):

∇·(ρu)=0

second equation is momentum conservation (Navier-Stokes equations):

ρ(∂u/∂t + (u·∇)u = -∇p + μ∇^2^u

where u is velocity vector, p is pressure, μ is dynamic viscosity and ρ is density.

c) Charge Conservation equation:

This equation describes the conversation of energy:

∇·$\vec{k}$ = 0

$\vec{k}$ = -σ∇φ

where ∇ is the divergence operator, $\vec{k}$ is the total density vector (A m^-2^), σ is the effective ionic conductivity of the electrolyte (S m^-1^) and ∇φ is the gradient of the electric potential.

The coupled solution of the above equations yields the distributions of the electrical field, fluid velocity, and concentration field.[S8]

Finite element simulation was employed to numerically simulate the diffusion of LiPSs starting from an initial concentration of 100 mol m^-3^ . The diffusion process was governed by Fick's law:

∂c/∂t + ∇∙(−D∙∇c) = R

where c is the concentration, D the diffusion coefficient, and R the reaction rate. Since no reactions occur apart from diffusion, R was set to 0. Simulations track the evolution of LiPSs concentrations in single-shell and double-shell structures on time scales. [S9]

**Supplementary Figures and Tables**


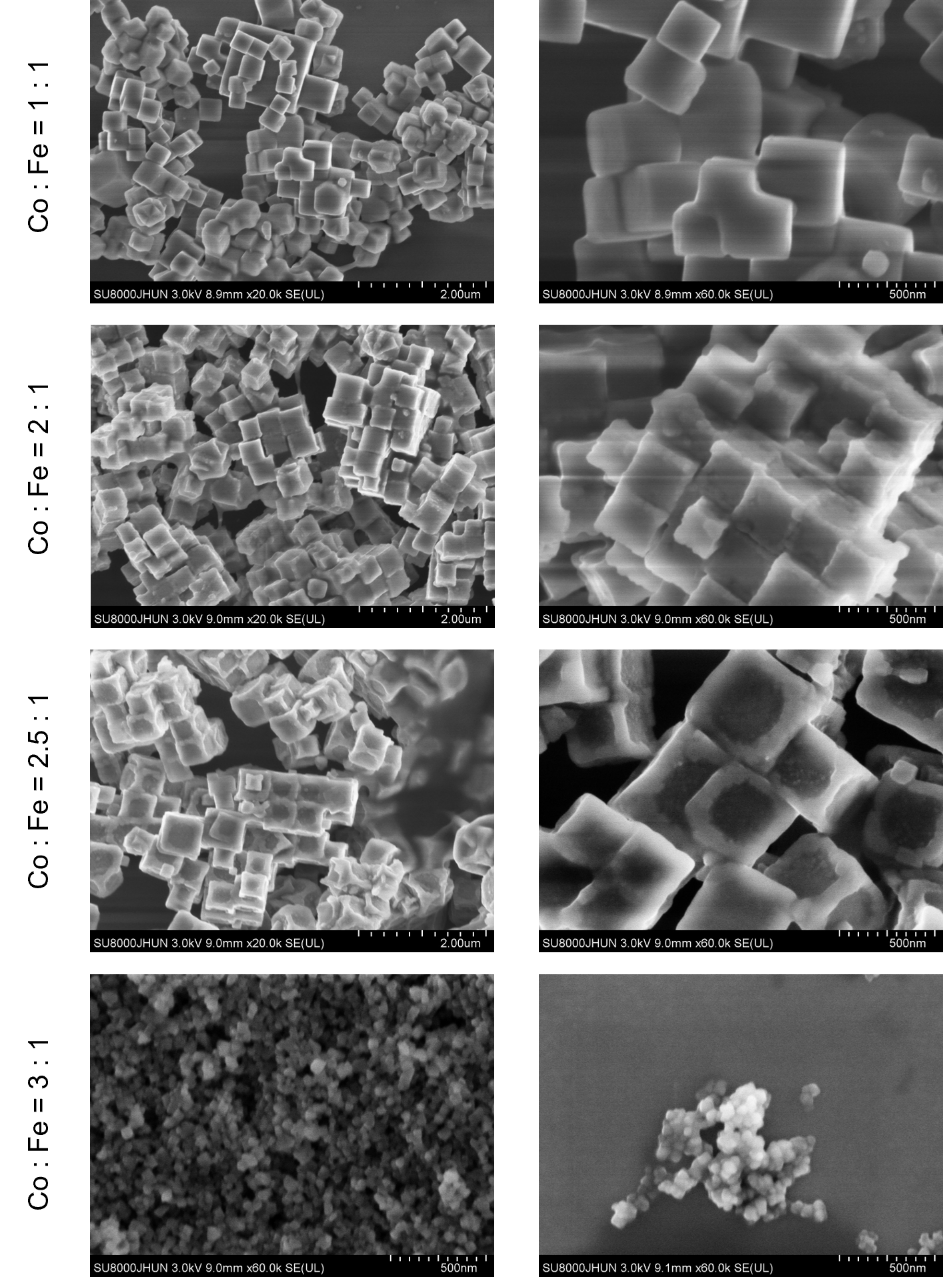


# Fig. S1 Morphology of CoFePBA with different Co/Fe ratios observed under SEM.

# Fig. S2 XPD spectra of CoFePBA with different Co/Fe ratios.


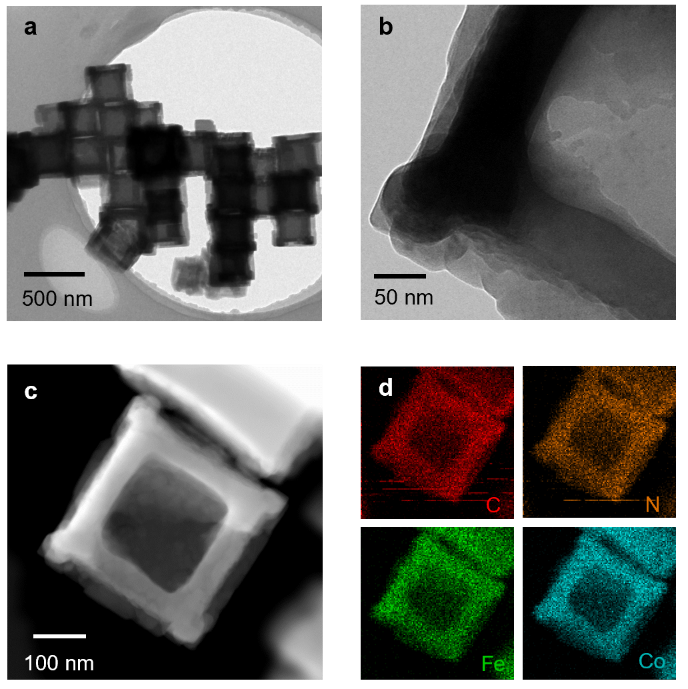


# Fig. S3 Morphology and elemental EDS mapping of Co_2.5_FePBA under high-resolution TEM.


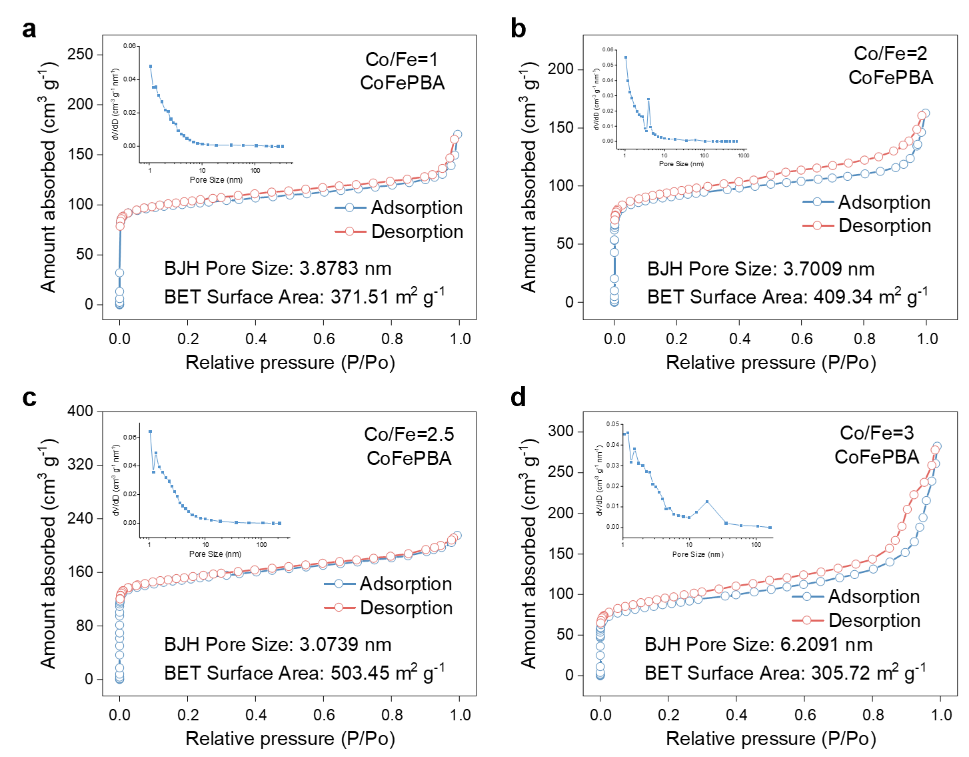


# Fig. S4 BET specific surface area and pore volume tests of CoFePBA with different Co/Fe ratios.


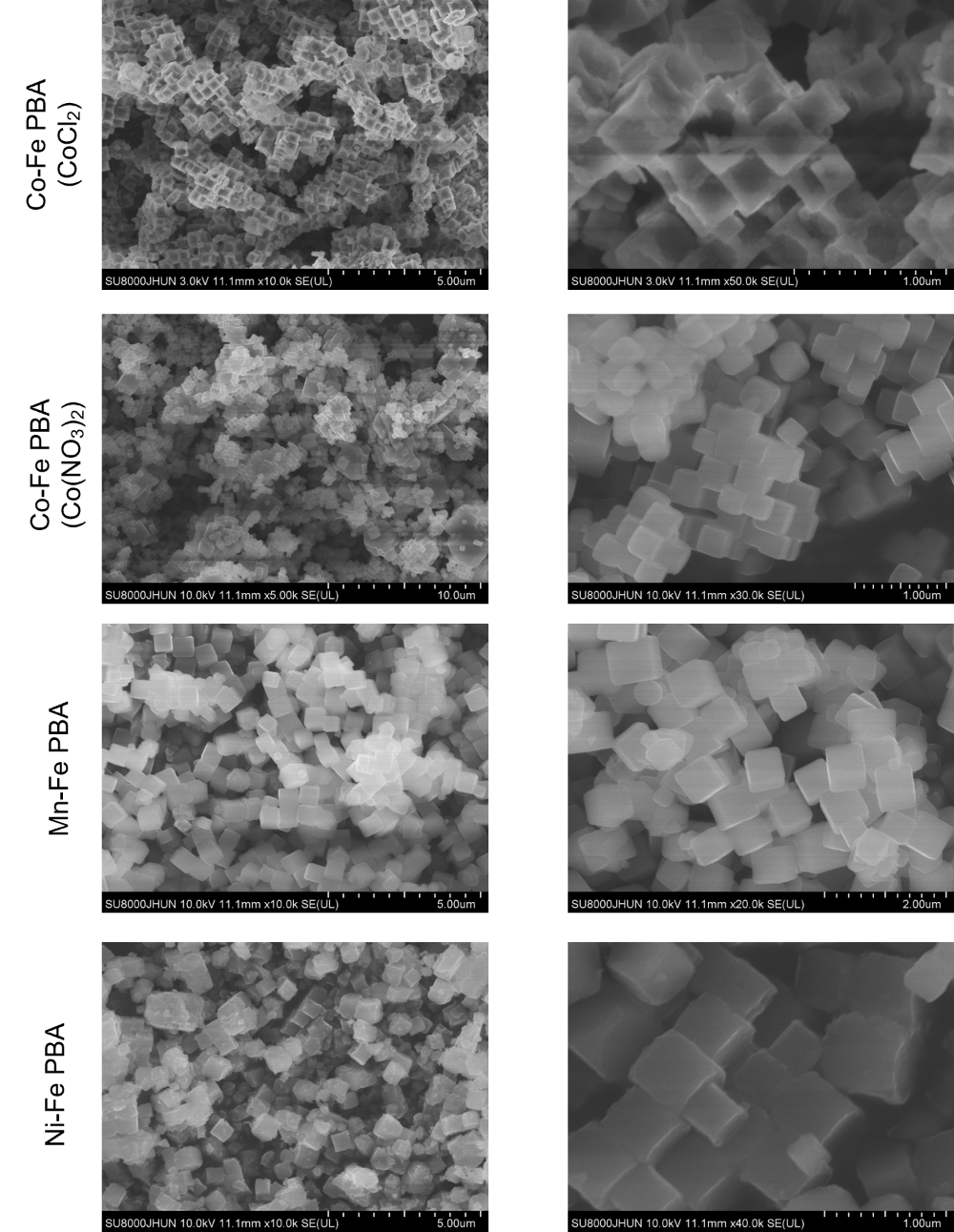


# Fig. S5 Morphology of bimetallic PBAs prepared with different metal salts.


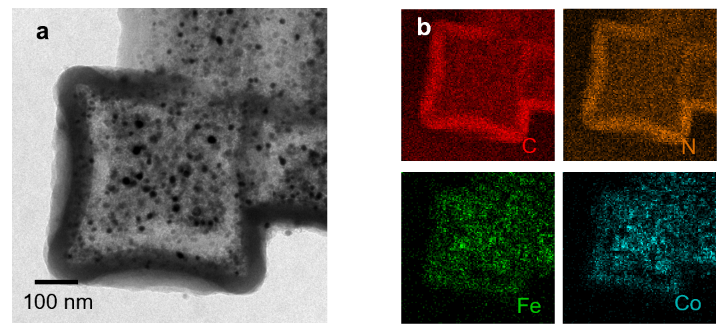


# Fig. S6 Morphological structure and EDS mapping of Co_2.5_Fe/NC cathode.


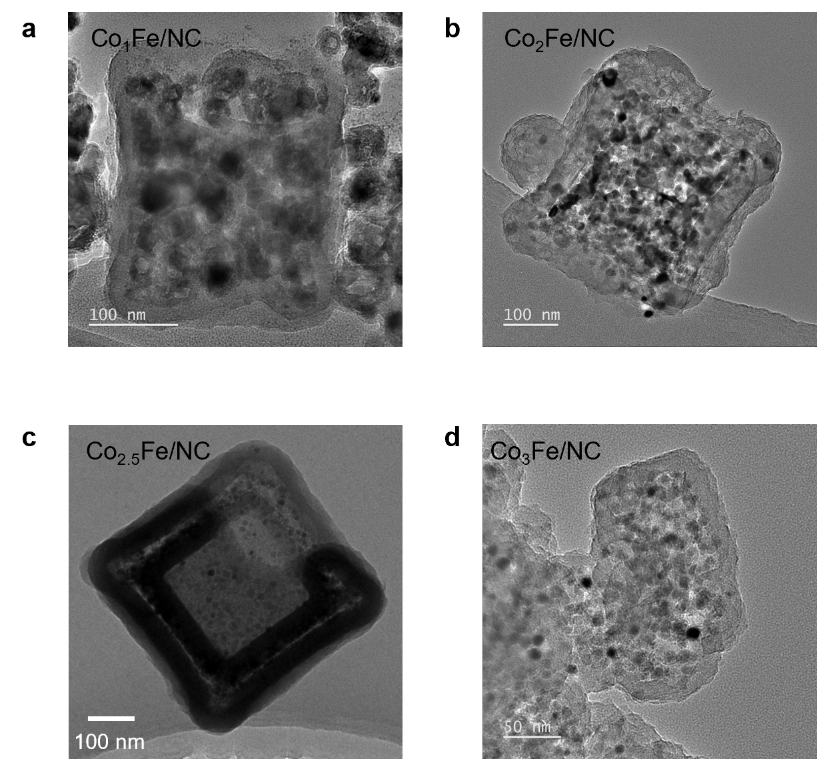


# Fig. S7 Morphology of CoFe/NC cathode with different Co/Fe ratios.


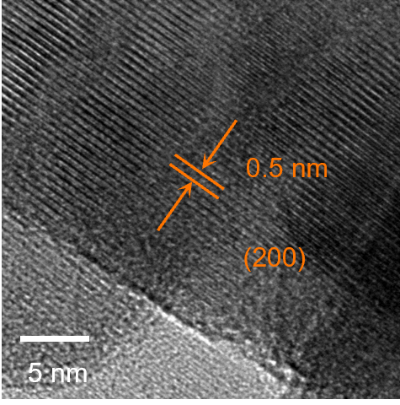


**Fig. S8** High-resolution TEM of the (200) crystalline surface of CoFe alloy.


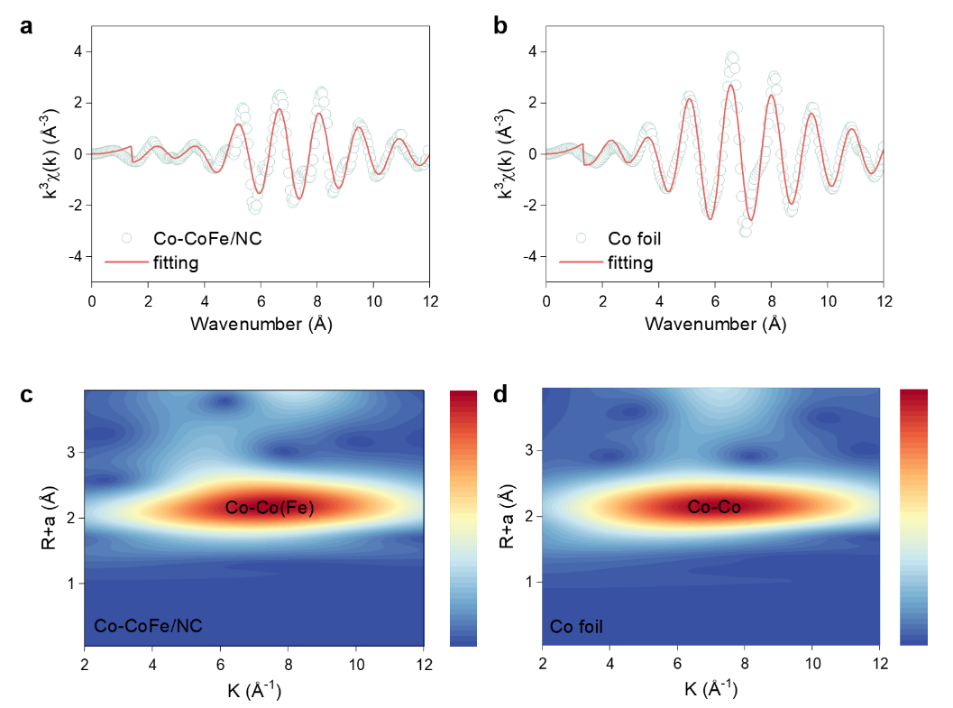


**Fig. S9** K-edge k-space experimental EXAFS spectra and fitting curves of Co in CoFe/NC (a) and Co foil (b). c, d) Wavelet transforms of k^2^-weighted EXAFS spectra at the Co K-edge of CoFe/NC (c) and Co foil (d).


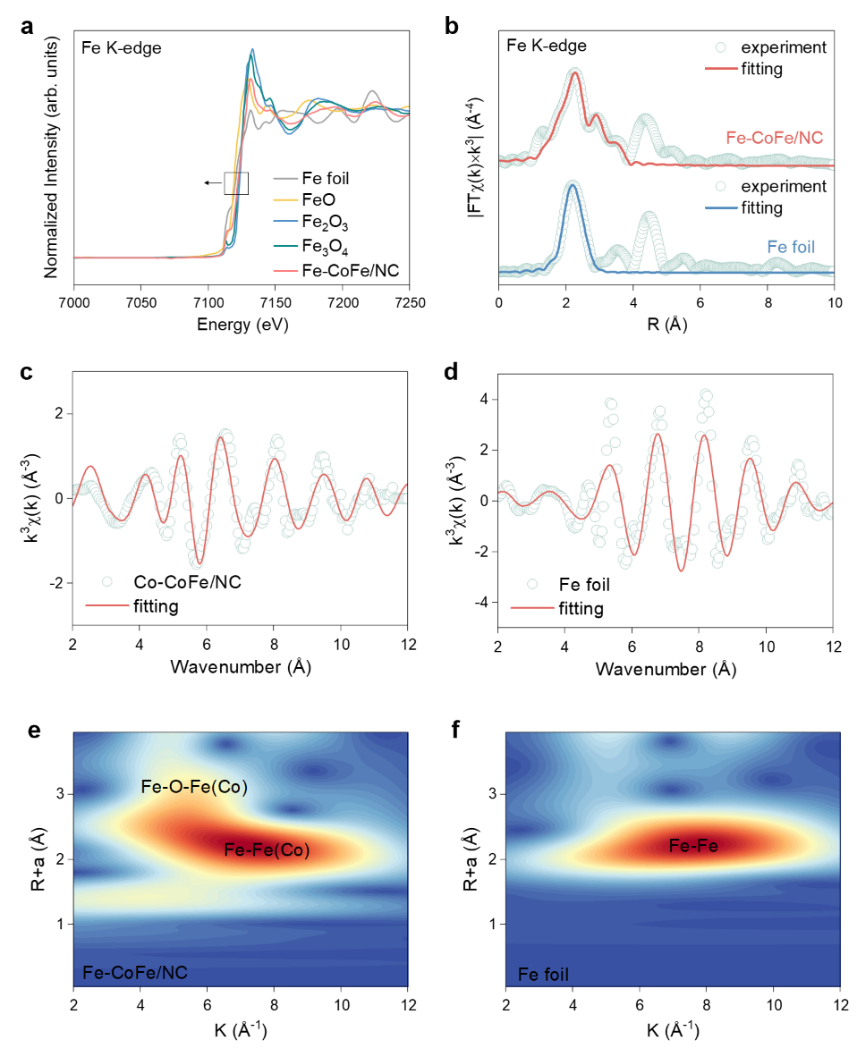


**Fig. S10** a) Fe K-edge XANES of CoFe/NC. b) Fourier transform magnitude of Fe K-edge EXAFS spectrum of Co_2.5_Fe/NC and Co foil. c, d) K-edge k-space experimental EXAFS spectra and fitting curves of Fe in CoFe/NC (c) and Fe foil (d). e, f) Wavelet transforms of k^2^-weighted EXAFS spectra at the Fe K-edge of CoFe/NC (e) and Fe foil (f).


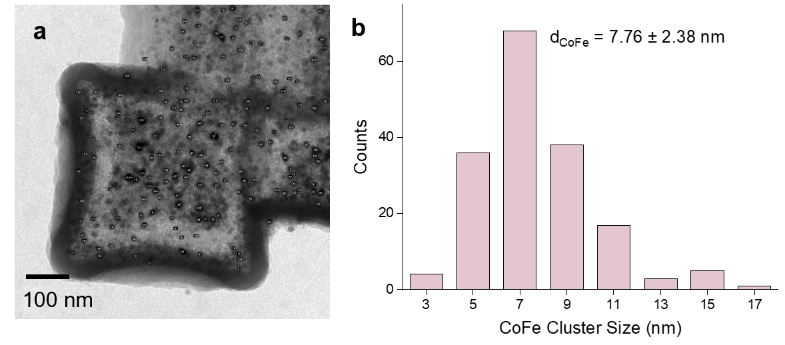


# Fig. S11 Particle size statistics of CoFe alloy nanoparticles in CoFe/NC.


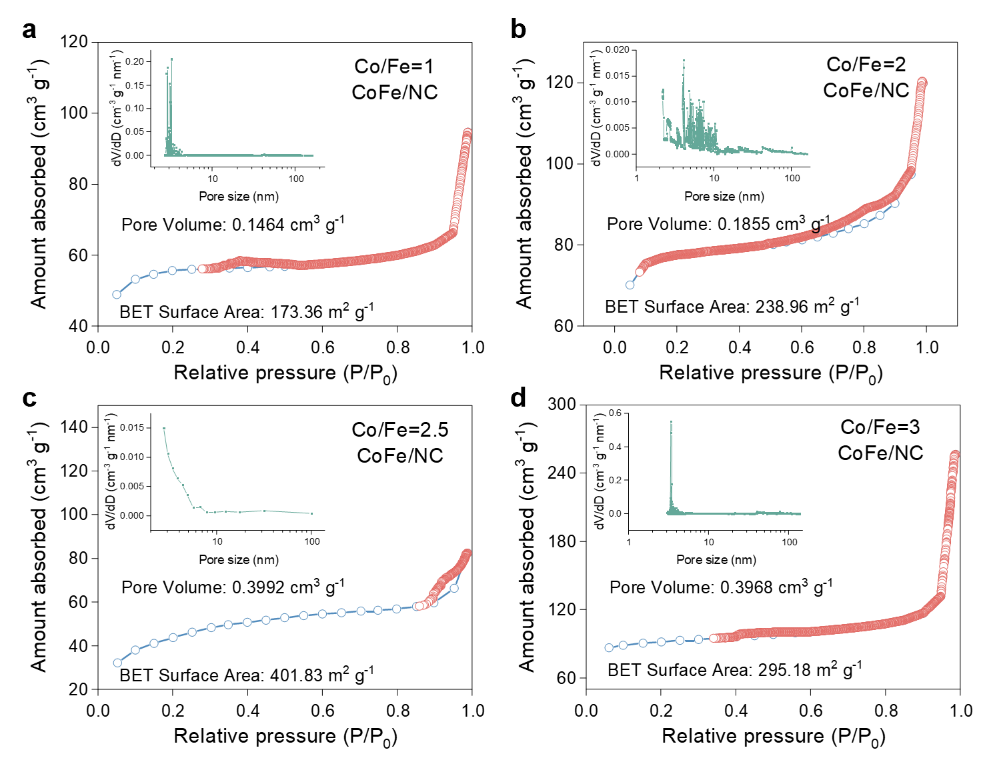


# Fig. S12 BET specific surface area and pore volume tests of CoFe/NC with different Co/Fe ratios.

# Fig. S13 Thermogravimetric analysis curve of CoFe/NC@S cathode.


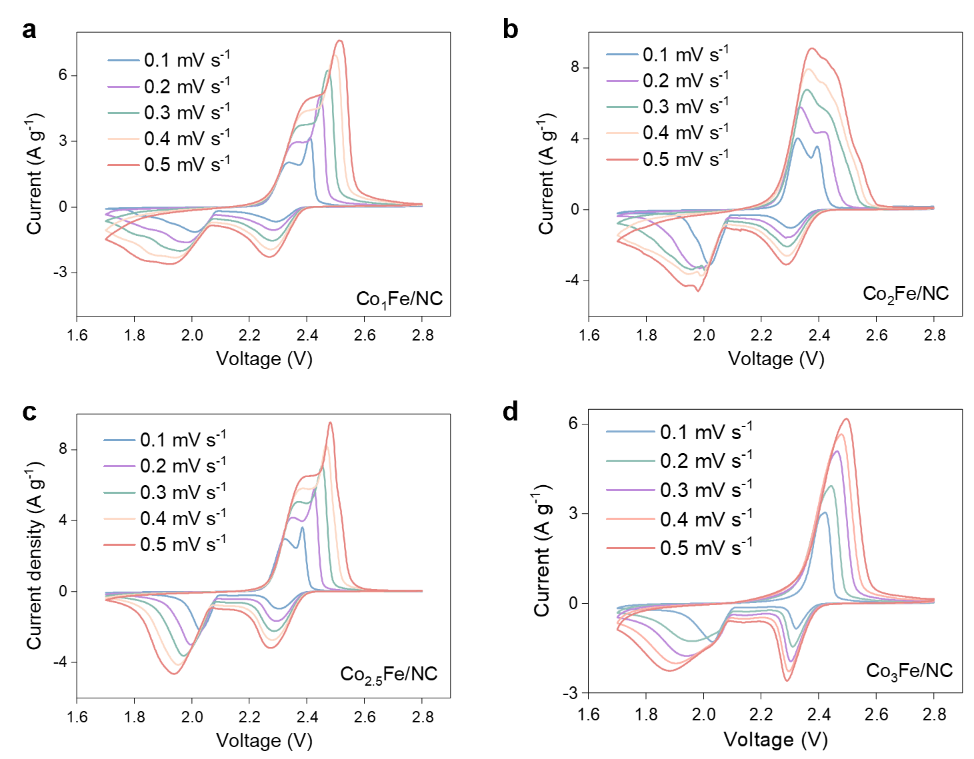


# Fig. S14 CV curves for different cathodes at different sweep speeds.

# Fig. S15 Contour plot of CV patterns for Co_1_Fe/NC.

# Fig. S16 The Li-ion diffusion coefficients for the conversion of Li_2_S_n_ to Li_2_S.

# Fig. S17 Binding energies with Li_2_S_4_ at different sites on CoFe alloys.


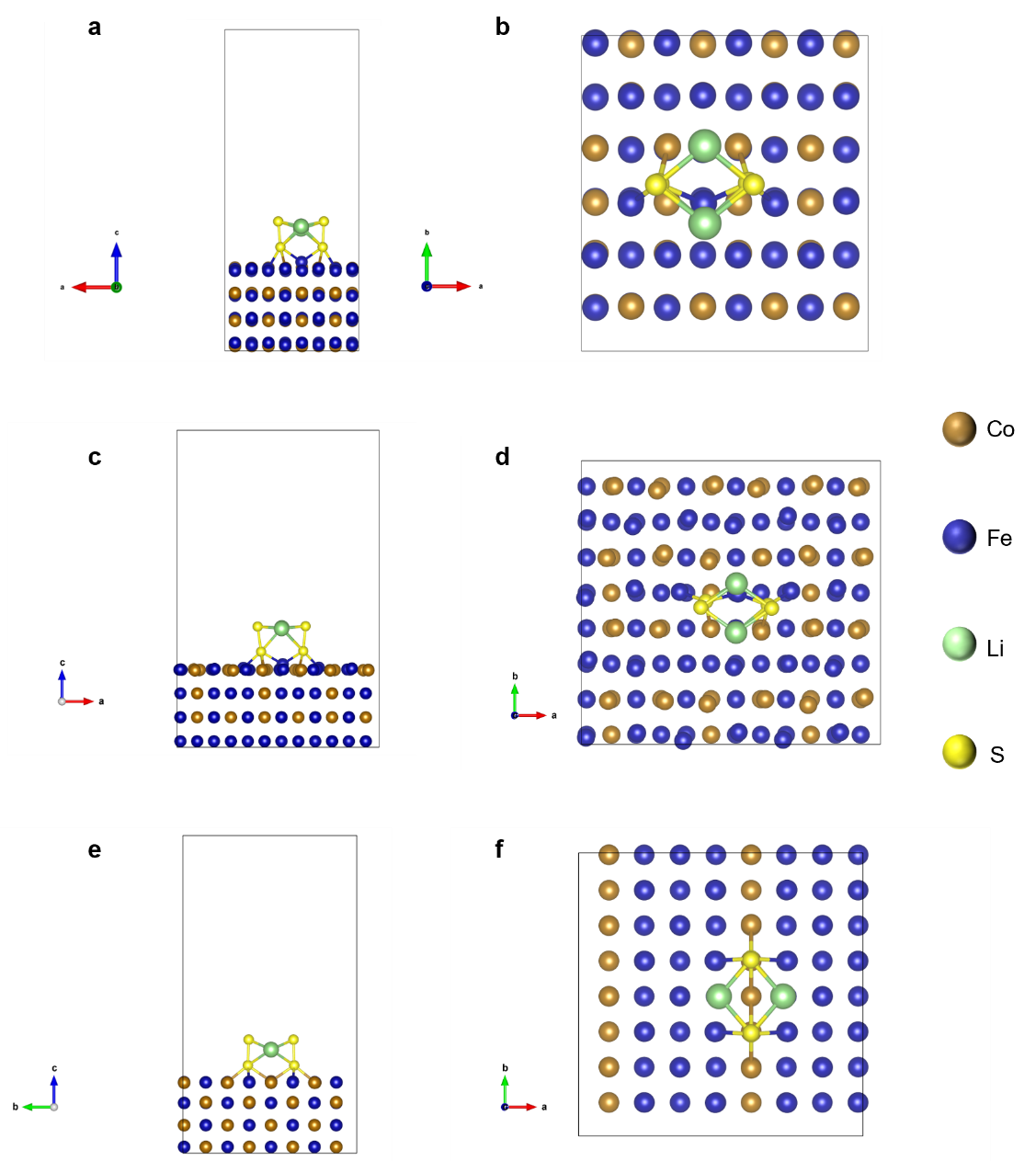


# Fig. S18 Modeling of Li_2_S_4_ adsorption on different ratios of CoFe alloys.


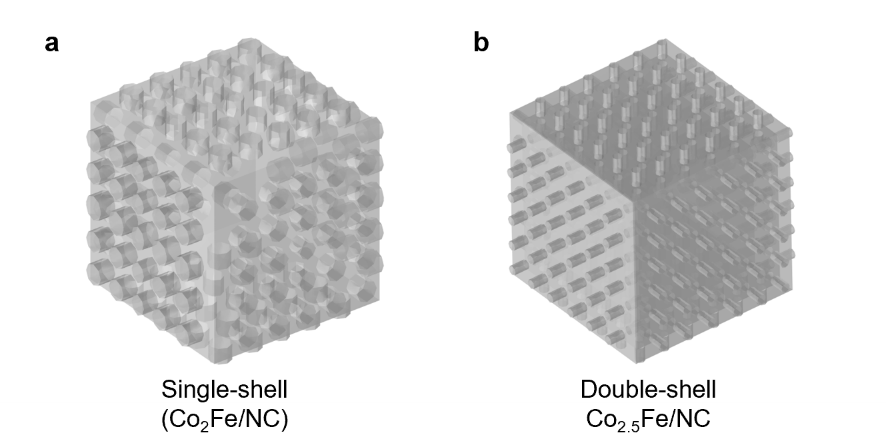


# Fig. S19 Finite element simulation modeling of single-shell Co_2_Fe/NC and double-shell Co_2.5_Fe/NC nanocubes.


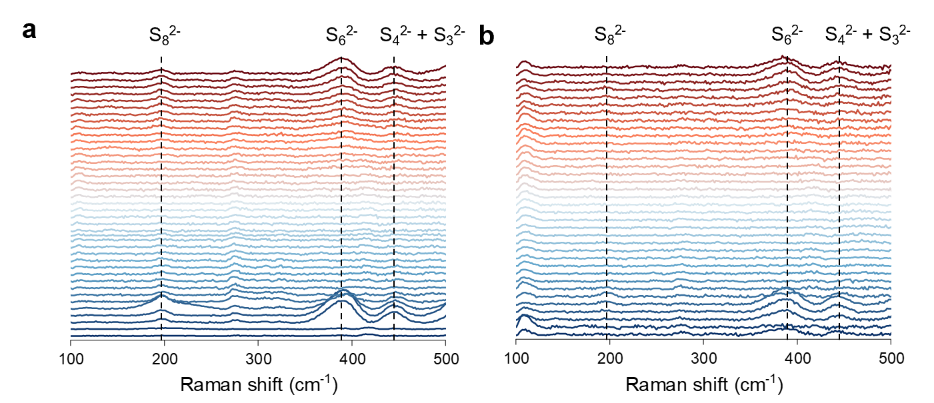


# Fig. S20 In situ Raman test patterns of Co_2_Fe/NC and Co_2.5_Fe/NC cathodes.


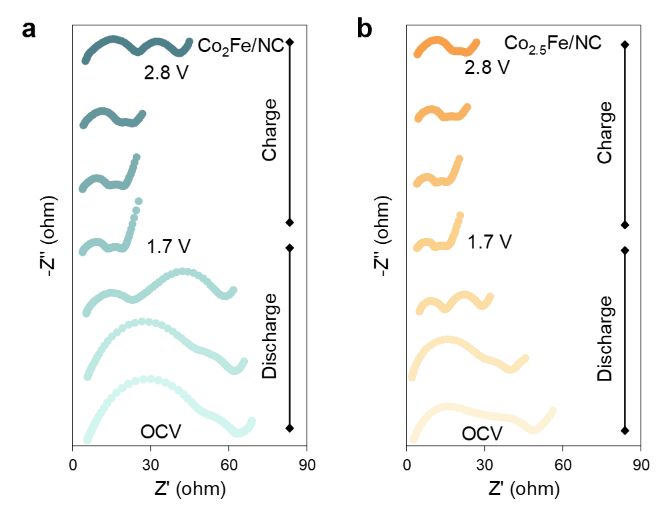


# Fig. S21 In situ EIS test spectra of Co_2_Fe/NC and Co_2.5_Fe/NC cathodes.


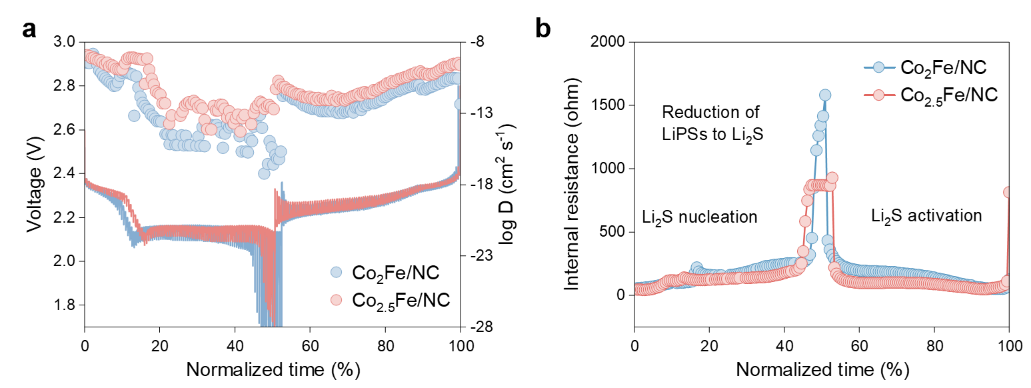


# Fig. S22 GITT test curves of Co_2_Fe/NC and Co_2.5_Fe/NC cathodes.

# Fig. S23 Potentiostatic discharge profiles of Li_2_S nucleation.

# Table S1 Comparison of the cycling stability and rate capability of the Co_2.5_Fe/NC cathode with those reported in other studies

| S Cathode | Number of cycles | Capacity decay per cycle | Rate capability | Refs. |
| --- | --- | --- | --- | --- |
| Co_2.5_Fe/NC | 1000 (2C) | 0.0156% | 676 mAh g^-1^  (4C) | This Work |
| PB@6h sulfur host | 500 (0.2C) | 0.0235% | 510 mAh g^-1^  (2C) | [S10] |
| P-FeTe_2-x_@NC | 1000 (1C) | 0.031% | 681 mAh g^-1^  (3C) | [S11] |
| Poled BTO@S | 1000 (1C) | 0.036% | 590 mAh g^-1^  (1C) | [S12] |
| S@Zr-MTAC | 1000 (2C) | 0.037% | 856 mAh g^-1^  (4C) | [S13] |
| CoNi@C-CNFs | 500 (2C) | 0.071 | 705 mAh g^-1^  (3C) | [S14] |

# Table S2 The mass for each component in the Ah-level Li-S batteries

| Compoent | Mass (g) |
| --- | --- |
| Cathode | 1.56 |
| Anode | 0.43 |
| Al foil | 0.19 |
| Separator | 0.08 |
| Electrolyte | 3.33 |
| Tab & package | 1.08 |
| Total | 6.67 |

**Supplementary References**

1. G. Kresse, J. Hafner, Ab initio molecular dynamics for open-shell transition metals. Phys. Rev. B **48**(17), 13115–13118 (1993). <https://doi.org/10.1103/physrevb.48.13115>
2. G. Kresse, J. Furthmüller, Efficient iterative schemes for *ab initio* total-energy calculations using a plane-wave basis set. Phys. Rev. B **54**(16), 11169–11186 (1996). <https://doi.org/10.1103/physrevb.54.11169>
3. J. Perdew, K. Burke, M. Ernzerhof, Generalized gradient approximation made simple. Phys. Rev. Lett. **77**(18), 3865–3868 (1996). <https://doi.org/10.1103/PhysRevLett.77.3865>
4. G. Kresse, D. Joubert, From ultrasoft pseudopotentials to the projector augmented-wave method. Phys. Rev. B **59**(3), 1758–1775 (1999). <https://doi.org/10.1103/physrevb.59.1758>
5. R. Dronskowski, P.E. Bloechl, Crystal orbital Hamilton populations (COHP): energy-resolved visualization of chemical bonding in solids based on density-functional calculations. J. Phys. Chem. **97**(33), 8617–8624 (1993). <https://doi.org/10.1021/j100135a014>
6. V.L. Deringer, A.L. Tchougréeff, R. Dronskowski, Crystal orbital Hamilton population (COHP) analysis as projected from plane-wave basis sets. J. Phys. Chem. A **115**(21), 5461–5466 (2011). <https://doi.org/10.1021/jp202489s>
7. S. Maintz, V.L. Deringer, A.L. Tchougréeff, R. Dronskowski, LOBSTER: a tool to extract chemical bonding from plane-wave based DFT. J. Comput. Chem. **37**(11), 1030–1035 (2016). <https://doi.org/10.1002/jcc.24300>
8. G. Zhang, W. Feng, G. Du, Y. Zhang, Y. Yang et al., Thermodynamically-driven phase engineering and reconstruction deduction of medium-entropy Prussian blue analogue nanocrystals. Adv. Mater. **37**(26), 2503814 (2025). <https://doi.org/10.1002/adma.202503814>
9. S. Chen, Z. Zhu, K. Song, H. Zhang, D. Luo et al., Engineering Cu/Ru heterointerface-shelled nanocavities by the kirkendall effect for highly efficient nitrate electroreduction to ammonia. J. Am. Chem. Soc. **147**(40), 36494–36507 (2025). <https://doi.org/10.1021/jacs.5c11097>
10. D. Jin, M.R. Krumov, R.M. Mandel, P.J. Milner, H.D. Abruña, Prussian blue analogue framework hosts for Li–S batteries. ACS Energy Lett. **9**(12), 5822–5829 (2024). <https://doi.org/10.1021/acsenergylett.4c02857>
11. S. Li, H. Yang, H. Tong, W. Xu, Z. Wang et al., Orbital-tailoring strategy *via* dual-defect engineering in P-FeTe_2-_*_x_*@NC synergizes polysulfide adsorption-conversion for lithium-sulfur batteries. Adv. Mater. **37**(47), e11910 (2025). <https://doi.org/10.1002/adma.202511910>
12. J. Jiang, J. Ontaneda, S. Pal, Z. Guo, C. Forrester et al., Enhanced polysulfide trapping in Li–S batteries by dipole alignment in ferroelectric BaTiO_3_. Energy Environ. Sci. **17**(17), 6291–6301 (2024). <https://doi.org/10.1039/D4EE01936A>
13. W. Meng, Y. Wang, X. Zhou, Z.-J. Chen, Y. Zhao et al., Azo-bridged metal-organic frameworks with robust Zr(6)-cluster nodes: a dual-functional design for suppressing polysulfide shuttling in lithium-sulfur batteries. J. Am. Chem. Soc. **147**(48), 44479–44491 (2025). <https://doi.org/10.1021/jacs.5c16469>
14. L. Ma, Q. Wu, M. He, J. Wu, B. Peng et al., Binary metal alloy electrocatalyst synergistically accelerates the bidirectional polysulfide conversions in lithium-sulfur batteries. Nano Lett. **25**(5), 1939–1947 (2025). <https://doi.org/10.1021/acs.nanolett.4c05606>
